# Crosstalk of Cancer Signaling Pathways by Cyclic Hexapeptides and Anthraquinones from *Rubia cordifolia*

**DOI:** 10.3390/molecules26030735

**Published:** 2021-01-31

**Authors:** Premalatha Balachandran, Mohamed Ali Ibrahim, Jin Zhang, Mei Wang, David S. Pasco, Ilias Muhammad

**Affiliations:** 1 National Center for Natural Products Research, Research Institute of Pharmaceutical Sciences, School of Pharmacy, University of Mississippi, Oxford, MS 38677, USA; jzhang3@olemiss.edu (J.Z.); meiwang@olemiss.edu (M.W.); dpasco@olemiss.edu (D.S.P.); 2 Natural Products Utilization Research Unit, Agricultural Research Service, United States Department of Agriculture, Oxford, MS 38677, USA

**Keywords:** *Rubia cordifolia*, cancer chemoprevention, anthraquinones, cyclic hexapeptides, signaling pathways, transcription factor

## Abstract

The anticancer activities of *Rubia cordifolia* and its constituents have been reported earlier, but their influence on the crosstalk of complex cancer-related signaling metabolic pathways (i.e., transcription factors; TF) has not yet been fully investigated. In this study, *R. cordifolia* root extract was subjected to the cancer signaling assay based bioactivity-guided fractionation, which yielded the following compounds viz., three anthraquinones, namely alizarin (**1**), purpurin (**2**), and emodin (**3**); two lignans, namely eudesmin (**4**) and compound **5**; and two cyclic hexapeptides, namely deoxybouvardin RA-V (**6**), and a mixture of **6**+**9** (RA-XXI). The structures of the isolated compounds were determined by NMR spectroscopy and HRESIMS. The isolated compounds **1**, **2**, **3**, **6,** and a mixture of **6**+**9** were tested against a panel of luciferase reporter genes that assesses the activity of a wide-range of cancer-related signaling pathways. In addition, reference anthraquinones viz., chrysophanol (**11**), danthron (**12**), quinizarin (**13**), aloe-emodin (**14**), and α-lapachone (**15**) were also tested. Among the tested compounds, the cyclic hexapeptide **6** was found to be very active against several signaling pathways, notably Wnt, Myc, and Notch with IC_50_ values of 50, 75, and 93 ng/mL, respectively. Whereas, the anthraquinones exhibited very mild or no inhibition against these signaling pathways. Compound **6** being the most active, we tested it for stability in simulated intestinal (SIF) and gastric fluids (SGF), since the stability in biological fluid is a key short-coming of cyclic hexapeptides. The anticancer activity of **6** was found to remain unchanged before and after the treatment of simulated gastric/intestinal fluids, indicating that RA-V was stable. As a result, it could be bioavailable when orally used in therapeutics and possibly a drug candidate for cancer treatment. The mechanism for the preferential inhibition of these pathways and the possible crosstalk effect with other previously reported signaling pathways has been discussed.

## 1. Introduction

*Rubia cordifolia* Linn. (Fam. Rubiaceae) is commonly called *Indian Madder* or *Manjistha*. It is a perennial climber herb which can grow up to 19 ft. The genus *Rubia* consists of about 70 species worldwide, which are known to be among the first known species for their marked commercial and medicinal values [1]. Commercially, they were used as natural dyes for hair and food products, while in the folk medicine, they have been used as early as 2000 B.C. for the treatment of wounds, ulcers, skin disorders, rheumatism, and inflammation [1]. The plants in this family are known to produce anthraquinones (e.g., alizarin, purpurin, emodin), lignans, iridoids, oleananes, triterpenoids, and cyclic hexapeptides. Trihydroxy anthraquinone (purpurin) and xanthopurpurin-2-carboxylic acid (manjistin) are the two main components present in the root of *R. cordifolia* [2], in addition to 1,2-dihydroxyanthraquinone (alizarin). Alizarin, otherwise called mordant red, is the red dye derived from the root of *R. cordifolia*. Purpurin has been used in Chinese pharmacopeia to evaluate the quality of herbal medicine [3]. The *R. cordifolia* extract shows potent anticancer activity and this was the focus of many researchers over the years. Many studies have established the importance of quinones as potential antitumor agents, where alizarin was found effective in reducing His+ revertant and mollugin was also proven to potentiate autophagic activity, as well as induce growth inhibition and apoptosis of HN4 human oral cancer cells and SK-BR-3 breast cancer cells [1,4]. 1-Hydroxy-2-methylanthraquinone was another compound from the *R. cordifolia* root exhibiting cytotoxic effects on A375 malignant skin melanoma cells [1]. In 2008, Keun Son et al. published a study showing the anticancer activity of a number of anthraquinones isolated from the methylene chloride fraction of the roots of *R. cordifolia* L. [5]. Among the reported compounds, 1-acetoxy-3-methoxy-9,10-anthraquinone showed the strongest inhibitory activity towards DNA topoisomerase I [5].

Due to the potential antitumor activity of this genus, many research groups were interested in further exploring more active metabolites in this genus and interestingly, in 1977, Jolad et al. isolated the first two Rubiaceae-type cyclic hexapeptides from *Bouvardia ternifolia* (Rubiaceae); namely bouvardin and deoxybouvardin (RA-V) [6]. In 1983, Itokawa et al. isolated similar cyclic peptides from *R. cordifolia* and *R. akane,* while screening for anticancer compounds and named these peptides as Ras [7,8]. A total of approximately twenty-four cyclic hexapeptides have been identified from the *R. cordifolia* root so far and assigned names from RA-I to RA-XXIV. All these Rubiaceae cyclopeptides (RAs), which are mainly isolated from *Rubia* species, contained of 18-member ring and 14-member ring systems, and formed with one *α*-d-alanine, one *α*-l-alanine, three *N*-methyl-*α*-l-tyrosines, and one other proteinogenic *α*-l-amino acid, have shown potential antitumor activity [9]. The methanolic root extract of *R. cordifolia* has been shown to inhibit hepatocellular carcinoma cell proliferation and lactate dehydrogenase (LDH) release, and to increase lipid peroxidation (LPO) in a dose-dependent manner [10]. Most of the RAs compounds showed cytotoxicity against several cancer cells, including P-388 leukemia cells [11], A-549 human non-small cell lung carcinoma cells, and Hela cells [12].

Cancer is initiated and progressed by several genetic and epigenetic modifications that cause cells to proliferate and escape mechanisms that normally control their survival and migration. Several of these alterations are attributed to signaling pathways that control cell growth and division and can be placed in the context of distortion of wider signaling networks that fuel cancer progression. Many of these signaling pathways are hyperactivated in cancer conditions either due to the inactivation of tumor suppressor genes or mutations that covert proto-oncogenes to oncogenes [13]. Components of developmental pathways, such as Wnt, hedgehog, and notch can be affected in cancer, as can downstream nuclear targets of signaling pathways, Myc and NF-kB and these pathways crosstalk with each other. For example, the cytokine IL-6 activates STAT-3 signaling which stimulates the synthesis of Myc [14]. These developmental signaling pathways are crucial for stem and progenitor cell homeostasis and function, and their inhibition could be pursued as a strategy to control cancer progression in many cancer types [15]. Vega et al. [16] identified ten crucial signaling pathways that play a significant role in various stages of cancer development. These pathway alterations occur in at least 33 cancer types, 64 subtypes with identified patterns. During an extensive analysis of 9125 tumors by Vega et al. [16], 89% percent of tumors had at least one driver alteration in these pathways and 57% percent of tumors had one alteration potentially targetable by currently available drugs. Thirty percent of tumors had multiple targetable alterations indicating the importance of analyzing these signaling pathways in cancer drug discovery research.

Due to the emerging significance of metabolic signaling pathways, signal target drugs have been considered as potential candidates for cancer therapy. In 2002, Darnell has pointed out the list of transcriptions factors such as Myc, Stat3, Wnt, ETS, E2F, Smad, Notch, and NF-κB, which are overactive in certain types of cancers [17]. This approach has been supported by the presence of more human oncogenes in signaling pathways compared to the oncogenic transcription factors [18]. Due to the potential anticancer activity of *R. cordifolia* extract, and the significance of targeting cancer-related signaling pathways towards anti-cancer drug discovery, this current study was conducted to evaluate the influence of *Rubia* extract, its pure compounds (anthraquinones), and cyclic hexapeptides (deoxybouvardin RA-V (**6**), and a mixture of **6** and RA-XXI (**9**)) on cancer signaling pathways. In this paper, the mechanism for preferential inhibition of these pathways and the possible crosstalk effect with other previously reported signaling pathways has been discussed.

## 2. Results and Discussion

The 70% ethanolic extract of the *R. cordifolia* showed very potent inhibition of cancer signaling pathways with IC_50_ values ranging from 2.5 µg/mL for Wnt pathway to 25.6 µg/mL for Notch signaling (Table 1).

**Figure 1:**
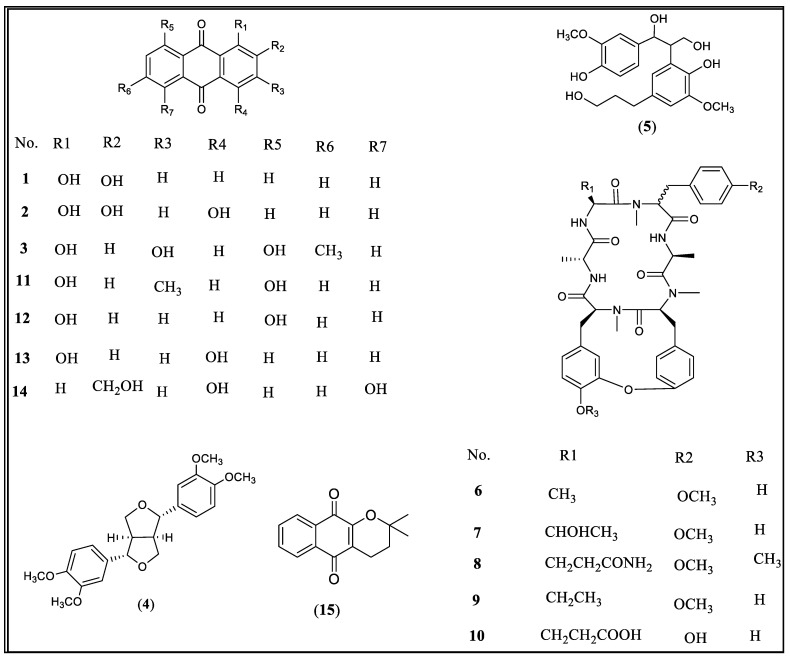
The chemical structures of compounds **1**–**15**.

The bioassay-guided fractionation, based on luciferase reporter gene assays, of the active root extract of *R. cordifolia*, using centrifugal preparative thin-layer chromatography (for details, see Materials and Methods), resulted in the isolation of three anthraquinones (**1**–**3**) and two lignans (**4** and **5**) (Figure 1).

Compounds **1**–**5** [alizarin (**1**), purpurin (**2**) and emodin (**3**), eudesmin (**4**) and 2-[2-hydroxy-5-(3-hydroxypropyl)-3-methoxyphenyl]-1-(4-hydroxy-3-methoxyphenyl)propane-1,3-diol (**5**)] have been isolated previously and their structures were suggested by comparison of their spectroscopic data with those published [19,20,21,22,23]. Subsequently, a mixture of cyclic hexapeptides have been isolated through the bio-activity guided fractionation and identified. From this mixture, the cyclic hexapeptides RA-V (**6**) and mixture **6**+**9** have been purified, and its HR-ESIMS has been acquired and compared with the reported compounds to confirm its chemical structure [6]. The activity of compound **6** has been tested against a panel of cancer-related signaling pathways using gene reporter assays, its activity is compared with a 70% ethanol extract and their IC_50_ values are reported in Table 1.

Compounds **1**–**3** were additionally purchased from commercial sources (Sigma-Aldrich, St. Louis, MO, USA) to supplement their low yield with purities of ≥97%, ≥95%, and ≥90%, respectively. In addition, to understand the structure activity relationship, five closely related anthraquinones reference compounds that previously reported from *R. cordifolia*, but were not isolated during the course of this study, were also purchased Sigma- Aldrich. These include chrysophanol (**11**), danthron (**12**), quinizarin (**13**), aloe-emodin (**14**), and α-lapachone (**15**) with purities between ≥96%–≥98%. Among these, *α*-lapachone (**15**) is the most active compound, which inhibited more than 50% of Stat3, Smad, NF-kB, Myc, ETS, and Wnt induction at 5 µM (Table 2). Among the anthraquinones tested at 30 µM, while **1** showed a selective inhibition of Wnt and Hedgehog signaling pathways, **3** inhibited the activation of several pathways with Wnt being the most sensitive. Compound **2** is the least active among these three anthraquinones (Table 2). The structure activity relationship of the anthraquinones suggested the increase of the inhibitory activity against STAT3, Myc, and Wnt with the increase of the number of electrons donating substituents. This was obvious via comparing the activities of compound **3** which has four electron donating groups versus the tested anthraquinones **1**–**2**, **11**–**13** which have either two or three electron donating groups. Compounds **11**–**14** did not show any activity towards these signaling pathways at the same concentration of 30 µM (Table 2).

Values are a percentage of luciferase induction at the given concentrations by the indicated inducers when compared to pTK as the control. Test agents were added to the cells 30 min before the indicated inducer and were harvested in the luciferase assay 4 or 6 (Notch, FoxO, Wnt, Hedgehog, and miR21) h later. No inducer was added to the cells transfected with the control vector (pTK), FoxO, and miR-21.

The cyclic hexapeptide **6** showed the most potent activity as it inhibited the activation of several pathways across the panel even at a very low concentration and IC_50_ values were less than 0.5 µg/mL (Table 1). Likewise, mixture **6**+**9** exhibited a potent activity across the panel of transcription factors, similar to **6**. Apart from compounds **6** and **6**+**9**, none of the compounds inhibited luciferase expression driven by the minimal thymidine kinase promoter (pTK, control vector) at the concentrations tested, indicating the lack of general cytotoxicity or luciferase enzyme inhibition (Table 1 and Table 2). For compound **6**, inhibition of pTK is much higher than other signaling pathways indicating its specific activity is more than its toxicity. The results depicted in Table 1 show that the anticancer potency of *R. cordifolia* has been largely contributed by the presence of **6** and **6**+**9** and its activity is mediated through the inhibition of several cancer signaling pathways with IC_50_ values of 0.2–5 µg/mL against most sensitive targets. Mishra et al. [24] investigated the effect of cyclic peptides on normal fibroblast cell lines and no cytotoxicity was observed up to 1000 µg/mL.

NF-kB has been reported previously as a molecular target for *Rubia’s* cyclic pepetide RA-V’s anticancer activity [25,26]. Compound **6** suppresses inflammation and tumor growth by targeting TAK1 and interrupting TAK-TAB2 interaction in the NF-kB signaling pathway [27]. Our results indicate that in addition to NF-kB, several other signaling pathways play a cumulative role for its activity and the Wnt pathway being more sensitive than NF-kB [25,26]. Compound **6** could also inhibit the PI3K/AKT pathway thus affecting cell survival and migration [28]. Yue et al. [29] have shown the anti-angiogenic potential of **6** through the ERK1/2 signaling pathway in HUVEC and HME-1 endothelial cells. They have indicated that **6** may regulate different signaling pathways in addition to ERK1/2 signaling. Our results show that it is true that the mechanism of action of **6** targets several interdependent signaling pathways across the panel of transcriptional factors and is not confined to any single signaling pathway/mechanism.

**Figure 2:**
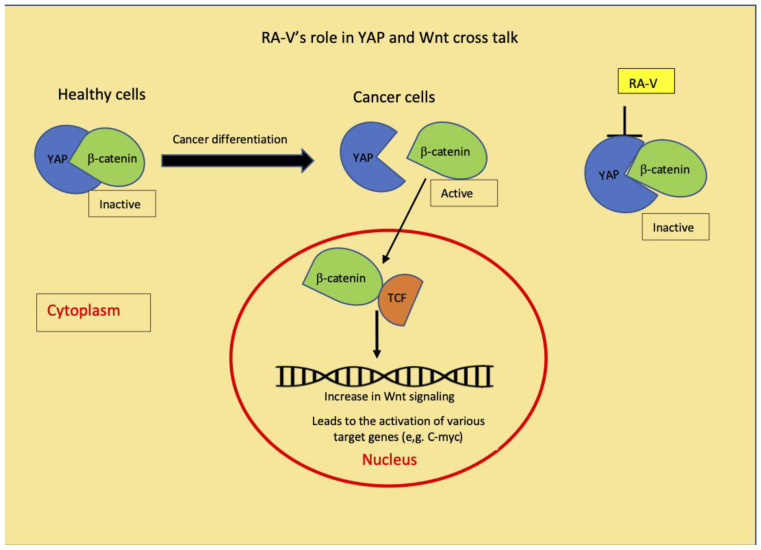
Schematic diagram depicting a crosstalk between Wnt and YAP pathways and how the YAP inhibition by RA-V could cause the inhibition of Wnt and decreases other signaling pathways observed in this study.

Recently, another molecular mechanism of compound **6** has been proposed by Ji et al. [30]. Compound **6** isolated from *Bourvardia ternifolia* has been shown to control tumor growth via the inhibition of YAP/TAZ activation. The yes-associated protein (YAP) and its paralog, PSD-95/Dlg/ZO-1 binding motif (TAZ) are transcription co-activators and effectors of the Hippo pathway and genetic inactivation of YAP could be an effective target to inhibit tumorigenesis [30]. Hippo signaling is an evolutionarily conserved pathway that controls organ growth by regulating cell proliferation, apoptosis, stem cell self-renewal, and dysregulation of this pathway contributes to the cancer development [31]. Ji et al. have mentioned that they are unsure about the reason for the RA-V specific inhibition of transcription vectors, e.g., YAP [30]. Our results indicate that Wnt signaling is most sensitive to the RA-V inhibition with IC_50_ values of 0.05 µg/mL. A recent review on YAP describes its crosstalk between Hippo and Wnt pathways. YAP preferentially acts as a transcriptional co-regulator of Hippo signaling pathway or as a regulator in the Wnt signaling pathway depending on the cell type [32]. The inhibition of YAP by RA-V, as demonstrated by Ji et al., could be responsible for the Wnt inhibition observed from our study. Therefore, our results throw more insight into the RA-V mechanism (Figure 2).

**Figure 3:**
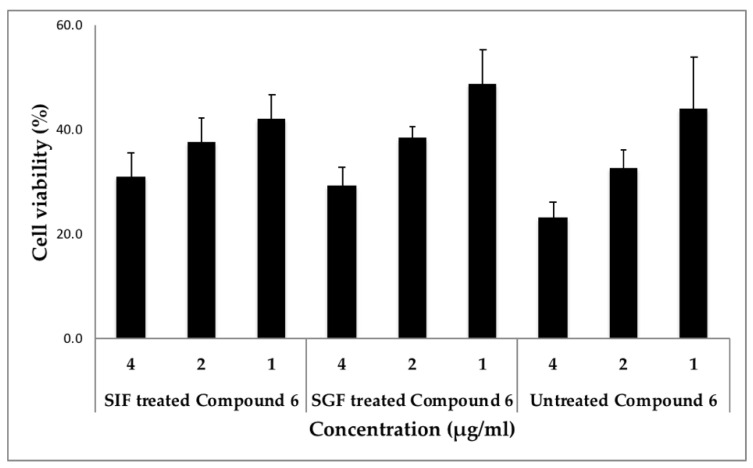
Stability of compound **6** in simulated intestinal and gastric fluids using T98G cells. Cells were incubated with different concentrations of compound **6** (Simulated Gastric Fluid or Simulated Intestinal Fluid treated or untreated) in triplicates and the cell viability was measured. Statistical significance was assessed using the unpaired *t*-test. All data were expressed as the mean + standard deviation (SD). The activities of SIF and SGF treated compound **6** were “non-significant” when compared to the untreated compound **6** at the concentrations tested.

**Figure 4:**
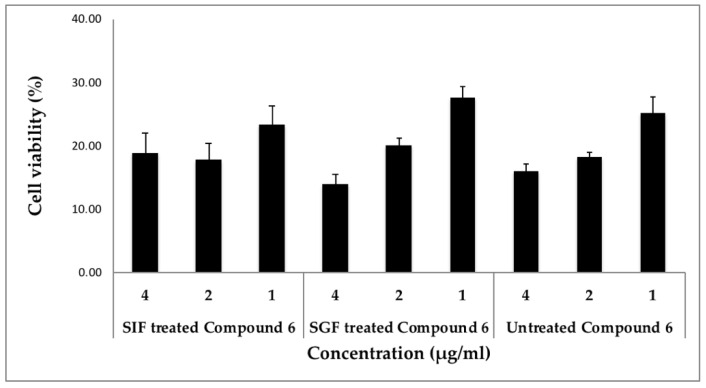
Stability of compound **6** in simulated intestinal and gastric fluids using MDA-MB231 cells. Cells were incubated with different concentrations of compound **6** (SGF or SIF treated or untreated) in triplicates and the cell viability was measured. Statistical significance was assessed using the unpaired *t*-test. All data were expressed as the mean + SD. The activities of SIF and SGF treated compound **6** were “non-significant” when compared to the untreated compound **6** at the concentrations tested.

Finally, the stability of digestion has been considered as a crucial physiochemical property to validate the use of such drugs in the biological system in order to evaluate their therapeutic value, since less is known about the stability of cyclic peptides from *Rubia*. We treated cyclic peptide (compound **6**) with either simulated gastric fluid (SGF) or simulated intestinal fluid (SIF), mimicking their exposure to biological fluids in humans and evaluated their activity in two different cancer cell lines compared to the untreated peptide. Our results are very promising as the potency of this cyclic peptide remained the same before and after the treatment with these simulated fluids in both T98G and MDA-MB 231 cells (Figure 3 and Figure 4). The treated and untreated compound **6** activity variations are not statistically significant. In addition, when SGF treated peptide was screened through the panel of cancer signaling pathways, its activity was comparable to the untreated peptide (Table S1). The results from these stability studies confirm the stability of this peptide and its activity is not destroyed by digestive fluids.

## 3. Materials and Methods

### 3.1. General Experimental Procedures

NMR spectra were acquired on a Varian Mercury 400 MHz spectrometer at 400 (^1^H) and 100 (^13^C) MHz in CDCl_3_, using the residual solvent as an internal standard. Multiplicity determinations (DEPT) and 2D-NMR spectra (HMQC, HMBC, NOESY) were obtained using standard Bruker pulse programs. HRESIMS were obtained by direct injection using a Bruker Bioapex-FTMS and Bruker micrOTOF (Bruker Daltonics, Bruker Inc., Billerica, MA, USA) with electrospray ionization (ESI). UHPLC/APCI-MS was performed using an Agilent 1290 UHPLC system coupled with an Agilent 6120 single quadrupole mass spectrometer. TLC was conducted on pre-coated silica gel 60 F_254_ (EMD Chemicals Inc., Darmstadt, Germany). Centrifugal preparative TLC (CPTLC, using a Chromatotron^®^, Harrison Research Inc., Palo Alto, CA, USA; model 8924, tagged with a fraction collector) was carried out on a 6 mm silica gel P_254_ (Analtech, Newark, DE, USA) disk. Samples were dried using a Savant Speed Vac Plus SC210A concentrator. The compounds were visualized by spraying the TLC plates, with 1% vanillin-H_2_SO_4_ as a spray reagent. The reference compounds **1**–**3** were purchased from and the reference standard doxorubicin (purity ≥ 98.0% pure) was procured from Sigma-Aldrich.

### 3.2. Plant Material

The grinded root plant material of *Rubia cordifolia* was purchased commercially from Vadik Herbs Inc. lot HG 071512, EXP 12/14, 5040 Commercial Circle, Suite F Concord, CA 94520, USA, in 2013.

### 3.3. Extraction and Isolation

The air-dried powdered root (40 g) was soaked in 95% EtOH (200 mL × 4 × 24 h each). The combined extract was filtered and dried (775 mg). The extract was dissolved in (MeOH:H_2_O; 90:10) then partitioned with *n*-hexane (200 mL × 4 × 24 h each). The aqueous layer has been separated from the *n*-hexane layer and dried (575 mg). The resulted two layers have been tested where the *n*-hexane was inactive, while the aqueous layer showed a potent activity. The aqueous extract was dissolved in (MeOH:H_2_O; 90:20) then partitioned with DCM (200 mL × 4 × 24 h each). The aqueous layer has been separated from the DCM layer and dried (70 mg). The resulted two layers have been tested where the DCM was active, while the remaining aqueous layer was inactive. The DCM extract has been subject to a LC analysis, where a considerable number of peaks has been detected, which encouraged us to do a larger scale up isolation using the powdered root (220 g). The 200 g has been exposed to a similar extraction protocol to yield 1 g DCM extract. The DCM extract (800 mg) was subjected to centrifugal preparative TLC (CPTLC, Chromatotron^®^), using a 6-mm silica gel rotor, the rotor was mounted on a Chromatotron^®^. The sample was dissolved in DCM and applied to the rotor under a rotation of 300 rpm, and then the rotor was left to dry. The dried rotor was eluted with *n*-hex:DCM, followed by DCM:MeOH mixtures in a polarity increasing order which afforded a number of fractions. The resulted fractions were monitored and pooled by the TLC analysis (silica gel; solvents: *n*-Hex-EtOAc; 7:3, and CHCl_3_-MeOH; 8:2) to yield a total of 33 fractions. The pooled fractions have been submitted to testing, where the main activity showed up in the late polar fractions. A detailed chromatographic separation, using CPTL of the less active, low polar fractions, Fr. 339–404, Fr. 405–416, and Fr. 516–517 led to the isolation of compounds **1**–**5** (20–30 mg each). A further separation of the highly potent polar fraction, Fr 563–603 (80 mg), by preparative HPLC (Luna C_8_ column; 250 × 150 mm, Phenomenex, Inc., Torrance, CA, USA), using H_2_O:CH_3_CN; 90:10→100% CH_3_CN (45 min, λ 220 nm) as a solvent yielded **6** (1.5 mg), followed by mixture **6**+**9** (2 mg). Compound **6** has been shown to be the cyclic hexapeptide RA-V with the formula C_40_H_48_N_6_O_9_ and *m*/*z* of 756.84, commonly called deoxybouvardin, which is known for its antitumor activity. While **9** has been shown to be RA-XXI which belongs to the homologous series with the difference of only one additional methylene group from **6**, with the formula C_41_H_50_N_6_O_9_ and *m*/*z* 770.87. The small difference in structure between **6** and **9** explains their co-elution upon chromatographic separation via HPLC and their similar activity pattern. The structures of compounds **1**–**6** and **9** were determined by using full NMR and HR-ESIMS data (please refer to Supplemental Information, Figures S1–S21).

### 3.4. UHPLC/APCI-MS Analysis

The analysis was performed on an Agilent 1290 Infinity liquid chromatograph coupled with an Agilent 6120 single quadrupole mass spectrometer. The LC column was a Waters Acquity UPLC^TM^ BEH RP-C_18_ column (1.7 µm, 2.1 × 150 mm). The mobile phase consisted of A (acetonitrile with 0.05% formic acid) and B (water with 0.05% formic acid) at a flow rate of 0.2 mL/min. The gradient elution started with 55% A for 8 min and then it was increased linearly to 100% A in 9 min, and held for 3 min. The column temperature was maintained at 30 °C. The compounds of interest were analyzed by ESI and APCI in both the positive and negative modes. The APCI positive mode was selected for analysis since it produced a better ion signal for the test compounds than the other ionization mode. The drying gas flow was 10 L/min and the nebulizer pressure was 30 psi. The drying gas temperature and vaporizer temperature were set to 250 and 200 °C, respectively. The capillary voltage was 3000 V and the corona current was 4.0 µA.

### 3.5. Transfection and Luciferase Assays 

Hela cells from ATCC were plated in white opaque 384 well plates at a density of 4300 cells/well in 30 µL of a growth medium (DMEM with 10% FBS and 1% Pen/step). The next day, the medium was aspirated and replaced with DMEM containing 10% FBS. The cells were transfected with respective plasmids using the X-tremeGENE HP transfection reagent (Roche). Luciferase vectors used in this assay are summarized in Table S2, Supporting Information. After 24 h of transfection, the test agents were added to the transfected cells, followed 30 min later by an inducing agent (IL-6 for Stat 3, TGF-beta for Smad, m-wnt3a for Wnt and PMA for AP-1, NF-kB, E2F, Myc, ETS, Notch and Hedgehog). No inducer was added for FoXO, miR-21, and PMA. After 4 h or 6 h of induction, the cells were lysed by the addition of the One-Glo luciferase assay system (Promega, Madison, WI, USA). The light output was detected in a Glomax Multi+ detection system with the Instinct Software (Promega, Madison, WI, USA). This luciferase assay determines if the test agent was able to inhibit the activation of cancer-related signaling pathways. In the case of FoxO and mi-R21, the enhanced luciferase activity by the test agent was assessed [18,33].

### 3.6. Cytotoxicity Assay

T98G and MDA-MB 231 cells from ATCC, were plated in clear 384-well plates at an initial density of 1700 cells/well for T98G cells and 2500 cells/well of MDA-MB 231 cells in 40 µL of a growth medium (DMEM with 10% FBS and 1% Pen/step). The next day, the test agents were added at the specified concentration, the treatment continued for 48 h, and the cell viability was finally assessed using the WST-8 assay Cell Counting Kit from Bimake, according to the manufacturer’s instructions [34]. The results were measured by absorbance at 450 nm using the SpectraMax M5 plate reader (Molecular Devices). The cell viability was calculated in comparison to the DMSO control.

### 3.7. SGF Digestion Stability Assay 

The simulated gastric fluid was prepared as described in the United States Pharmacopoeia (USP, 1995) [35] and consists of 0.2% w/v of NaCl and 0.7% v/v of hydrochloric acid at pH 1.2. Aliquots (48 µL) of SGF were placed in 1.5-mL microcentrifuge tubes and incubated in a water bath at 37 °C [35]. In addition, 2 µL of compound **6** (10 mg/mL in DMSO) was added to each of the SGF vials to start the digestion reaction. The incubation was continued for 60 min and the digestion was stopped with 150 µL of ice-cold cell culture media. The digested samples were used for the cytotoxicity and luciferase assays at different concentrations and the activities were compared between the untreated and SGF treated peptide samples.

### 3.8. SIF Digestion Stability Assay 

The simulated intestinal fluid (USP XXII) [35] pH 7.5, was purchased from the Ricca chemical Company. Pancreatin was added to SIF at the concentration of 10 mg/mL. Aliquots (48 μL) of SIF containing pancreatin were placed in 1.5-mL microcentrifuge tubes and compound **6** (2 μL) at a concentration of 10 mg/mL was added to each of the microcentrifuge tubes to start the reaction. The samples were incubated at 37 °C for 8 h in a water bath. At the end of the incubation, the reaction was immediately stopped by heat inactivation through placing the tube in a boiling water bath for 10 min. The samples were then diluted using ice-cold cell culture media. The digested samples were used for the cytotoxicity assay at different concentrations and the activities were compared between the untreated and SIF treated peptide samples.

### 3.9. Statistical Analysis

Statistical analysis was performed using Graph Pad PRISM (GraphPad Software Inc., San Diego, CA, USA). Statistical significance was assessed using the unpaired *t*-test, *p* > 0.05 was considered non-significant.

## 4. Conclusions

This study showed that the potent anticancer potency of *R. cordifolia* has been mainly contributed by the presence of cyclic hexapeptide **6** and **6**+**9**, whose activity is mediated through the inhibition of several cancer signaling pathways. It further confirms that the anthraquinones play a minor role in the anticancer potential of *R. cordifolia* when compared to cyclic hexapeptides, as they exhibit less inhibition towards the cancer signaling pathways. The crosstalk between cancer signaling pathways plays several complex roles in cancer and the identifying targets modulating these pathways serve as the current focus on the cancer treatment approach. Among them, the Wnt signaling pathway is known for its involvement in cancer initiation and progression via the activation of β-catenin signaling [32]. It has been reported that the cytoplasmic YAP is involved in regulating the tissue differentiation and migration of tumor cells by binding to β-catenin [36]. YAP has a negative regulatory effect on the Wnt signaling [37] and YAP effectively modulates the transcriptional activation of Wnt/β-catenin signaling. In healthy cells, where the Wnt signaling pathway is inactive, YAP binds to the destruction complex together with β-catenin, keeping the β-catenin inactive [36,37,38]. In cancer cells, YAP and β-catenin dissociate from the complex and this releases the β-catenin from the YAP binding. β-catenin moves to the nucleus and binds with TCF in the nucleus, which then activates the TF factor and Wnt signaling pathway becomes active [39,40]. The YAP inactivation by compound **6** reported by Ji et al. [30] could be responsible for the Wnt inhibition and subsequent inhibition of various transcription factors, as depicted in Figure 2. Taken together, our data suggest that this novel idea of regulation of compound **6,** in crosstalk between YAP and Wnt pathways leading to the modulation of other signaling pathways (Figure 2), provide additional insight into the RA-Vs (**6**) existing mechanism and warrants further detailed research into this cyclic hexapeptide’s crosstalk mechanism.

## Figures and Tables

**Table 1:** Activity of *Rubia* extract and compounds **6** and **6**+**9** against cancer-related signaling pathways in HeLa cells. IC_50_ values are in µg/mL that inhibited luciferase induction by 50%. Test agents were added to cells 30 min before the addition of the indicated inducer and were harvested in the luciferase assay for 4 or 6 h (Notch, FoxO, Wnt, Hedgehog, miR-21) later. No inducer was added to the cells transfected with FoxO, miR-21, or pTK control vector. ND: No activity detected.

	Stat3/IL-6	Smad/TGF-beta	Ap-1/PMA	NF-κB/PMA	E2F/PMA	Myc/PMA	Ets/PMA	Notch/PMA	FoxO	Wnt/m-wnt 3a	Hdghog/PMA	miR-21	pTK
*Rubia* 70% ETOH extract	8.9	11.9	5.2	5.9	16.9	3.7	8.4	25.6	ND	2.5	5.3	25.4	ND
Compound **6**	0.18	0.3	0.22	0.17	0.36	0.075	0.18	0.093	0.1	0.05	0.2	ND	0.9
Compound **6**+**9**	0.05	0.11	0.05	0.05	0.19	0.012	0.062	0.13	0.47	0.0013	0.046	0.7	0.47

**Table 2:** Activity of pure compounds against cancer-related signaling pathways in HeLa cells.

Pure Compounds	Stat3/IL-6	Smad/TGF-β	Ap-1/PMA	NF-kB/PMA	E2F/PMA	Myc/PMA	Ets/PMA	Notch/PMA	FoxO/No Induction	Wnt/m-wnt 3a	Hdghog/PMA	mi-R21/No Induction	pTK/No Induction
Compound **1** (Alizarine red) (30µM)	81	109	87	60	172	61	66	106	69	46	57	141	116
Compound **2** (Purpurine) (30µM)	102	163	73	65	190	61	103	194	94	77	152	259	198
Compound **3** (Emodin ) (30µM)	48	46	45	45	144	46	53	424	112	11	42	309	144
Compound **11** (Chrysophanol) (30µM)	171	160	115	90	251	144	130	195	102	114	214	280	183
Compound **12** (Danthron) (30µM)	137	101	122	62	328	115	120	126	85	122	168	156	101
Compound **13** (Quinizarin) (30µM)	164	159	107	62	282	81	127	198	101	97	142	171	134
Compound **14** (Aloe-Emodin) (30µM)	132	78	138	70	178	95	128	157	109	129	99	167	136
Compound **15** (Dehydro-α-lapachone) (DAL) (5µM)	22	27	88	44	82	44	45	85	100	53	86	127	97

## Data Availability

Not applicable.

## Supplementary Materials

Figure S1:^1^H NMR spectrum of Alizarin (1); Figure S2:^13^C NMR spectrum of Alizarin (1); Figure S3: ^1^H NMR spectrum of Purpurin (2); Figure S4: ^13^C NMR spectrum of Purpurin (2); Figure S5: ^1^H NMR spectrum of Emodin (3); Figure S6: ^13^C NMR spectrum of Emodin (3); Figure S7: ^1^H NMR spectrum of Eudesmin (4); Figure S8: ^13^C NMR spectrum of Eudesmin (4); Figure S9: ^1^H NMR spectrum of Neolignan (5); Figure S10 13C NMR spectrum of Neolignan (5); Figure S11: ^1^H NMR of cyclic hexapeptide mixrture; RA-V (6) and RA-XXI (9); Figure S12: ^13^C NMR of cyclic hexapeptide mixrture; RA-V (6) and RA-XXI (9); Figure S13: COSY of cyclic hexapeptide mixrture; RA-V (6) and RA-XXI (9); Figure S14: HMBC of cyclic hexapeptide mixrture; RA-V (6) and RA-XXI (9); Figure S15: HSQC expansion of cyclic hexapeptide mixrture; RA-V (6) and RA-XXI (9); Figure S16: HSQC expansion of cyclic hexapeptide mixrture; RA-V (6) and RA-XXI (9); Figure S17: LC/MS spectrum of RA-V (6) and RA-XXI (9); Figure S18: Mass (+) spectrum of RA-V (6); Figure S19: Mass (+) spectrum of RA-XXI (9); Figure S20 1H NMR of cyclic hexapeptide RA-V (6); Figure S21: 13C NMR of cyclic hexapeptide RA-V (6); Table S1: Activities of SGF treated and untreated compound 6 against cancer related signaling pathways in Hela cells; Table S2: Details of plasmids used in transfection.

## Sample Availability

Samples of the compounds are not available from the authors.
