# Supplementary material for: Crosstalk of Cancer Signaling Pathways by Cyclic Hexapeptides and Anthraquinones from Rubia cordifolia
Source: Molecules. 2021 Jan 31;26(3):735. doi: 10.3390/molecules26030735 (PMC7866972; doi:10.3390/molecules26030735)
Supplement: Supplementary file 1 [file molecules-26-00735-s001.pdf]

Cross talk of cancer signaling pathways by cyclic hexapeptide and anthraquinones from *Rubia cordifolia*

Premalatha Balachandran[1,*], Mohamed Ali Ibrahim[1,*], Jin Zhang[1], Mei Wang[1,2], David S. Pasco[1] and Ilias Muhammad[1,*]

[1]National Center for Natural Products Research, Research Institute of Pharmaceutical Sciences, School of Pharmacy, University of Mississippi, University, MS 38677, USA. (jzhang3@olemiss.edu ; dpasco@olemiss.edu)

[2]Natural Products Utilization Research Unit, Agricultural Research Service, United States Department of Agriculture, University, Mississippi 38677, USA (meiwang@olemiss.edu)

***Correspondence:**

MI: milias@olemiss.edu; Tel: +01-662-915-1051

PB: prembala@olemiss.edu; Tel: +01-662-915-3463

MAI: mmibrahi@olemiss.edu; Tel: +01-662-915-1147

Supporting Information Table

Number	Details
Figure S1	^{1}H NMR spectrum of Alizarin (1)
Figure S2	^{13}C NMR spectrum of Alizarin (1)
Figure S3	^{1}H NMR spectrum of Purpurin (2)
Figure S4	^{13}C NMR spectrum of Purpurin (2)
Figure S5	^{1}H NMR spectrum of Emodin (3)
Figure S6	^{13}C NMR spectrum of Emodin (3)
Figure S7	^{1}H NMR spectrum of Eudesmin (4)
Figure S8	^{13}C NMR spectrum of Eudesmin (4)
Figure S9	^{1}H NMR spectrum of Neolignan (5)
Figure S10	^{13}C NMR spectrum of Neolignan (5)
Figure S11	1H NMR of cyclic hexapeptide mixrture; RA-V (6) and RA-XXI (9)
Figure S12	^{13}C NMR of cyclic hexapeptide mixrture; RA-V (6) and RA-XXI (9)
Figure S13	COSY of cyclic hexapeptide mixrture; RA-V (6) and RA-XXI (9)
Figure S14	HMBC of cyclic hexapeptide mixrture; RA-V (6) and RA-XXI (9)
Figure S15	HSQC expansion of cyclic hexapeptide mixrture; RA-V (6) and RA-XXI (9)
Figure S16	HSQC expansion of cyclic hexapeptide mixrture; RA-V (6) and RA-XXI (9)
Figure S17	LC/MS spectrum of RA-V (6) and RA-XXI (9)
Figure S18	Mass (+) spectrum of RA-V (6)
Figure S19	Mass (+) spectrum of RA-XXI (9)
Figure S20	^{1}H NMR of cyclic hexapeptide RA-V (6)
Figure S21	^{13}C NMR of cyclic hexapeptide RA-V (6)
Table S1	Activities of SGF treated and untreated compound 6 against cancer related signaling pathways in Hela cells.
Table S2	Details of plasmids used in transfection

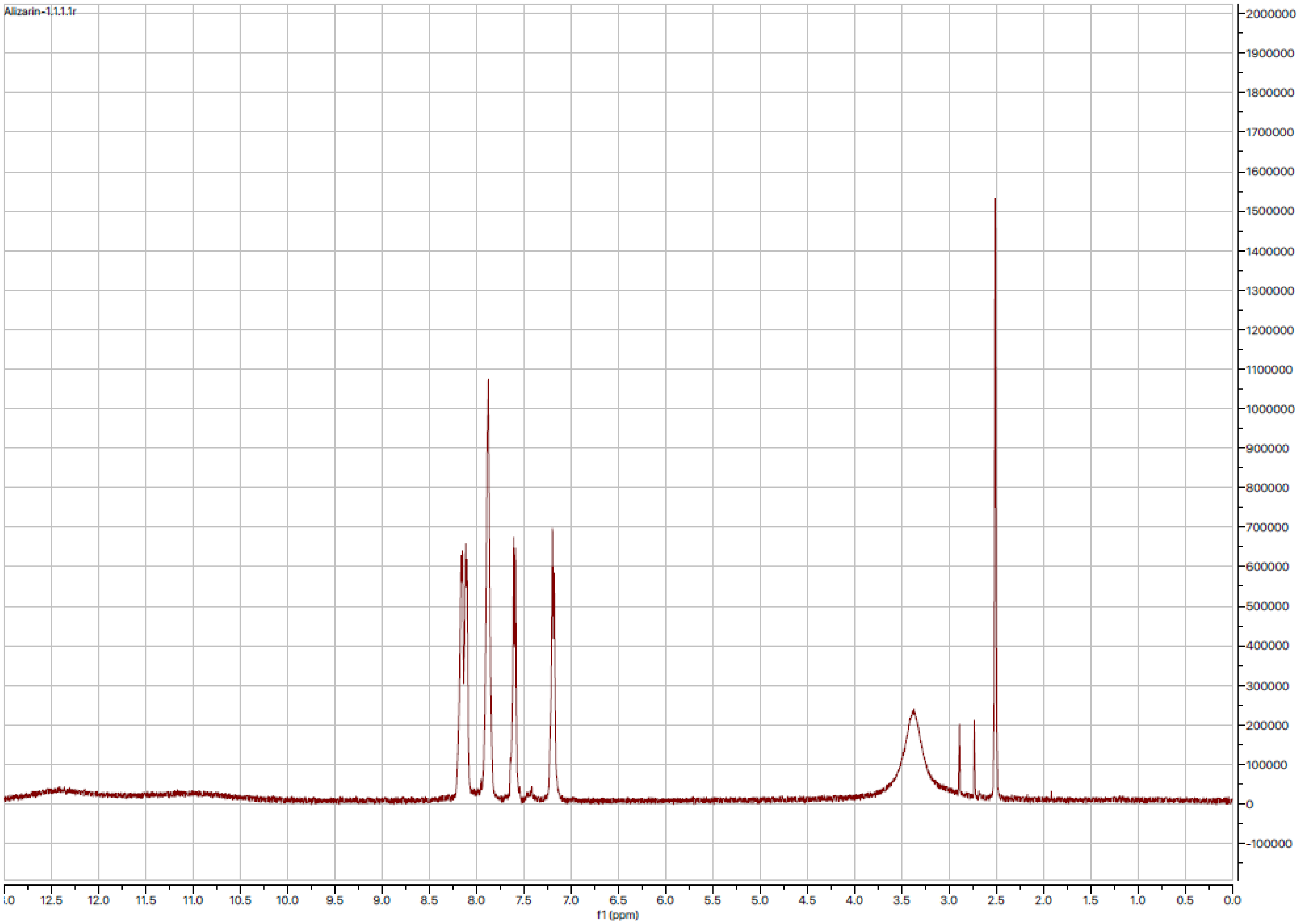

Figure S1: ^{1}H NMR spectrum of Alizarin (**1**) [400 MHz, DMSO-*d6*]

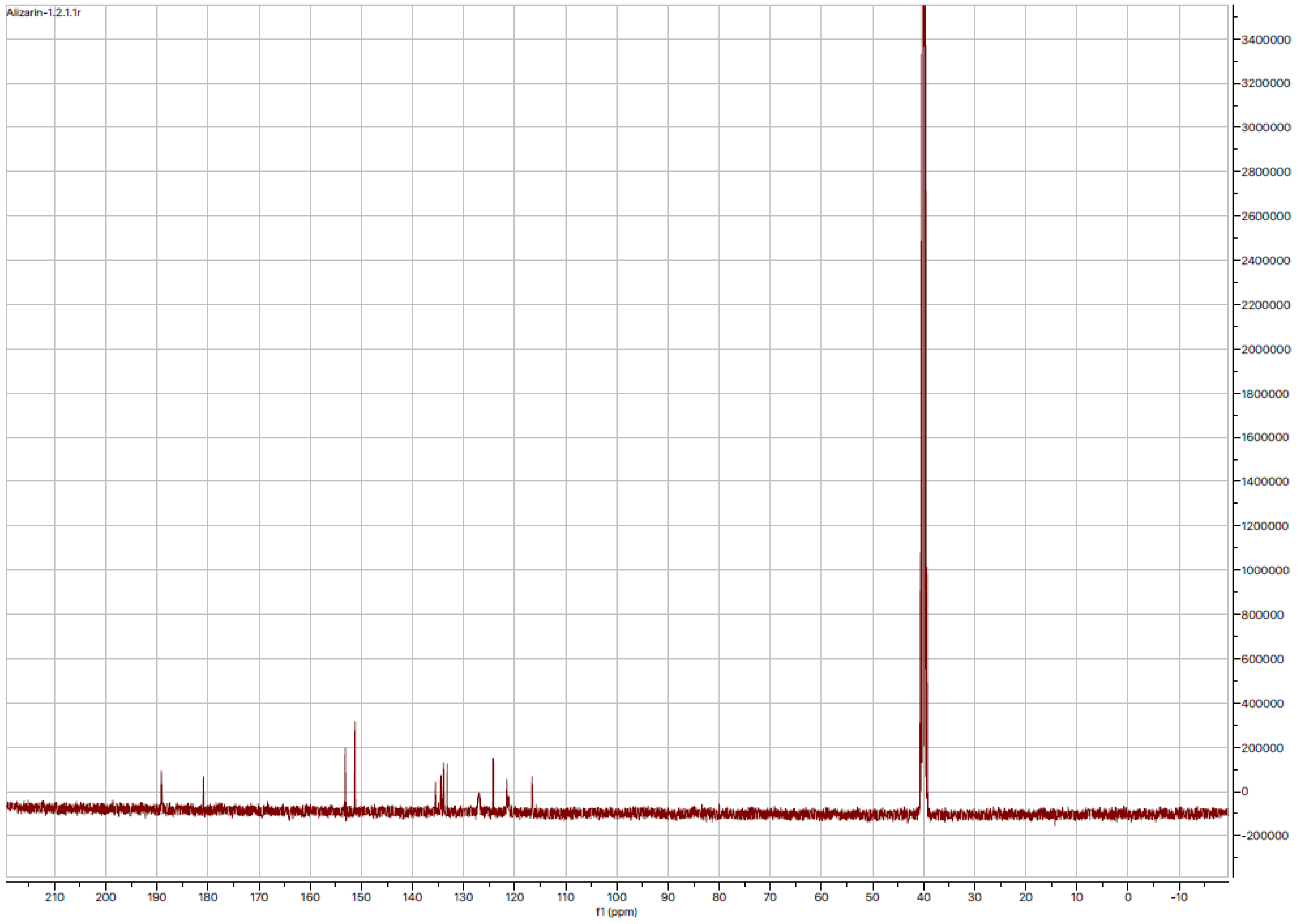

Figure S2: ^{13}C NMR spectrum of Alizarin (**1**) [400 MHz, DMSO-*d6*]

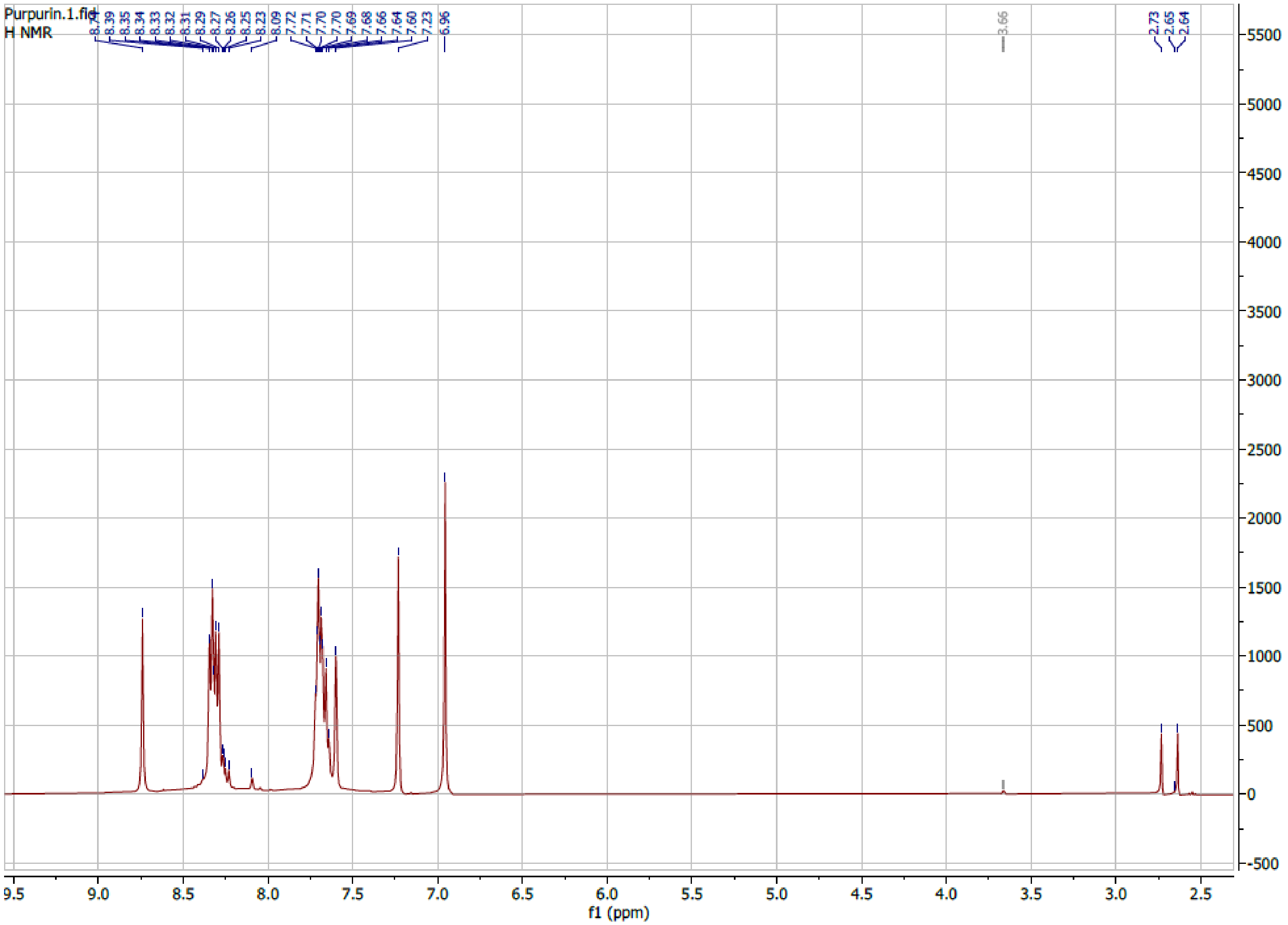

Figure S3: ^{1}H NMR spectrum of Purpurin (2) [400 MHz, Pyridine-*d5*]

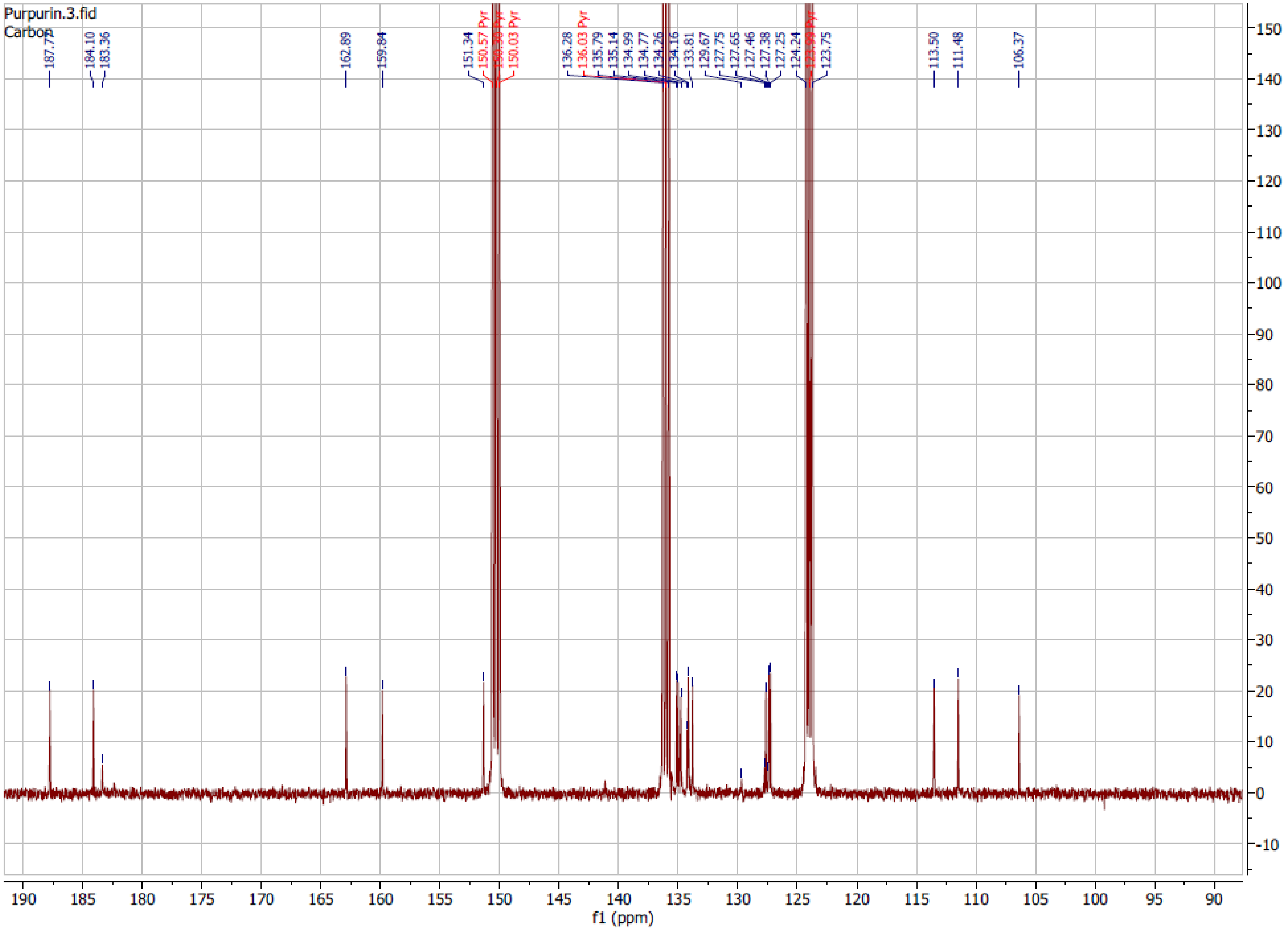

Figure S4: ^{13}C NMR spectrum of Purpurin (2) [400 MHz, Pyridine-*d5*]

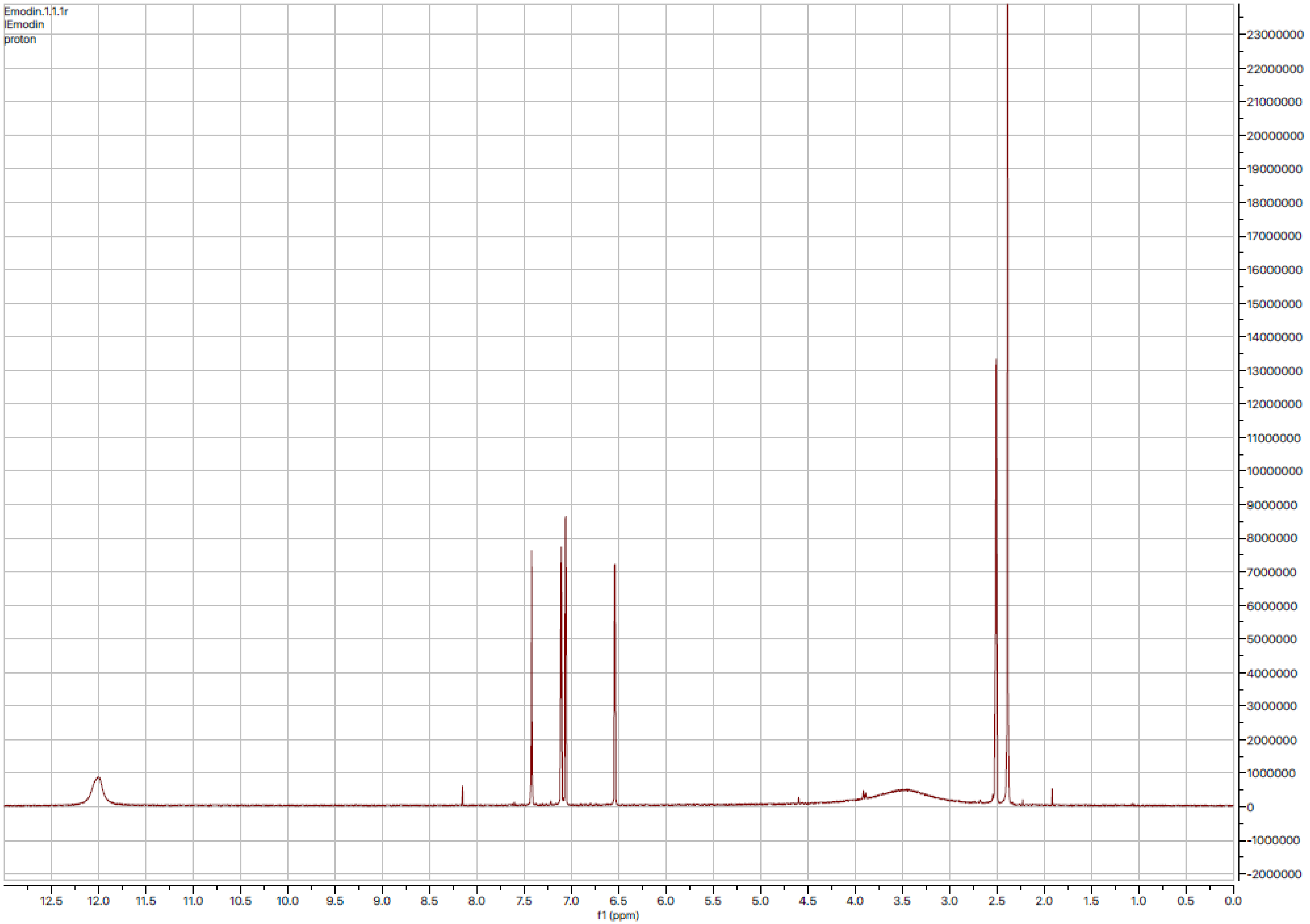

Figure S5: ^{1}H NMR spectrum of Emodin (**3**) [400 MHz, DMSO-*d6*]

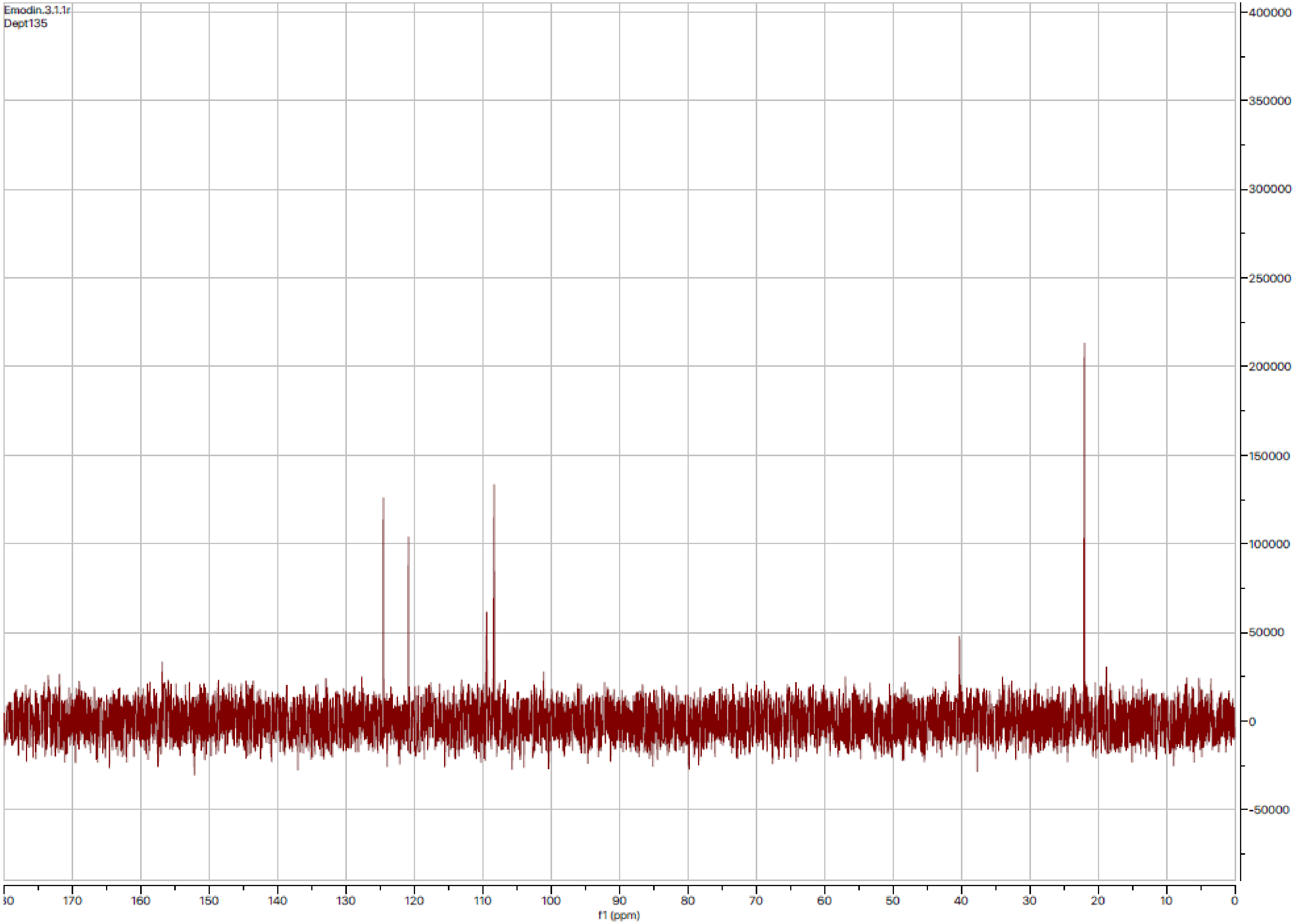

Figure S6: ^{13}C NMR spectrum of Emodin (3) [400 MHz, DMSO-*d6*]

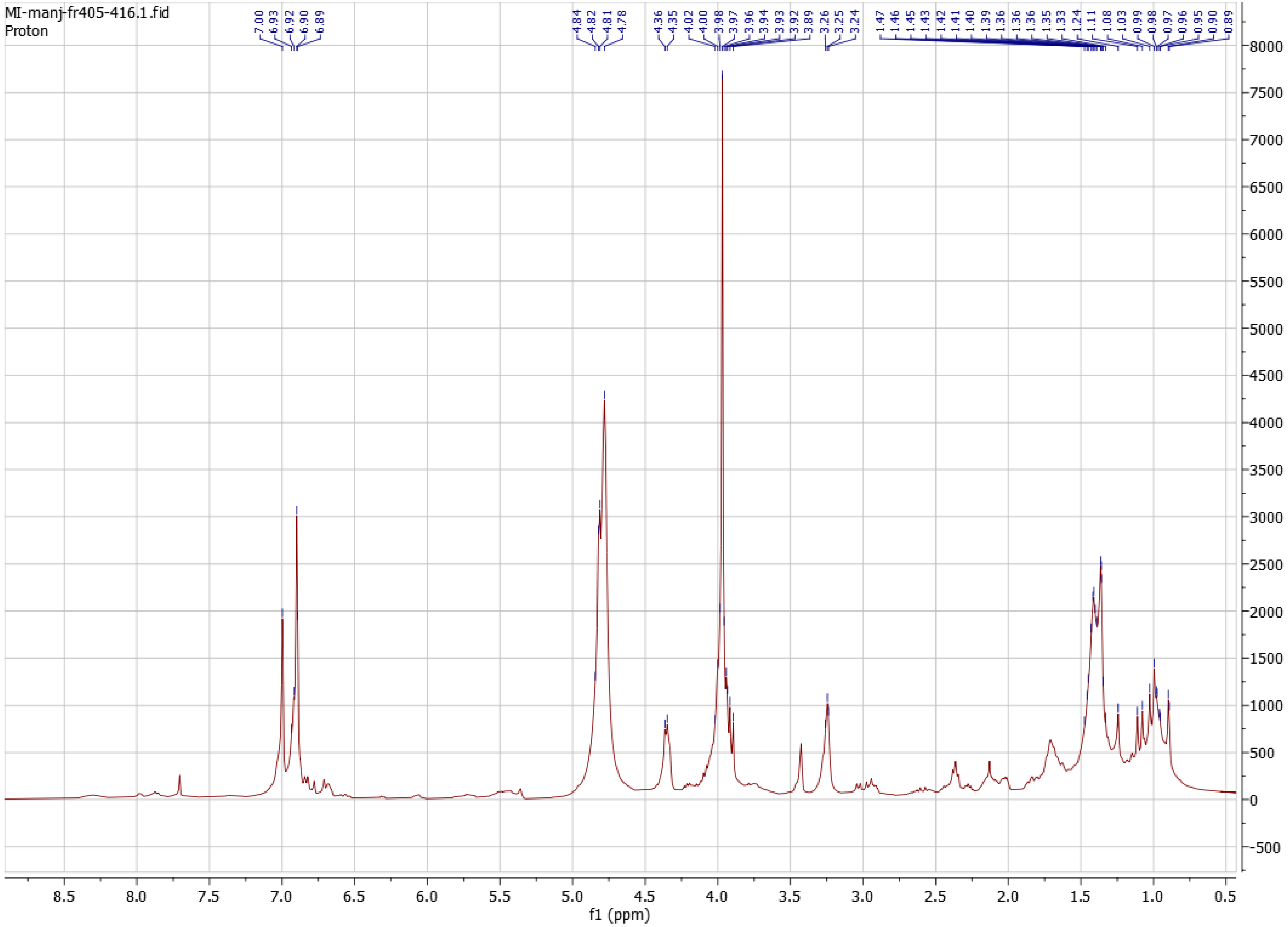

Figure S7: ^{1}H NMR spectrum of Eudesmin (4) [400 MHz, MeOH-*d4*]

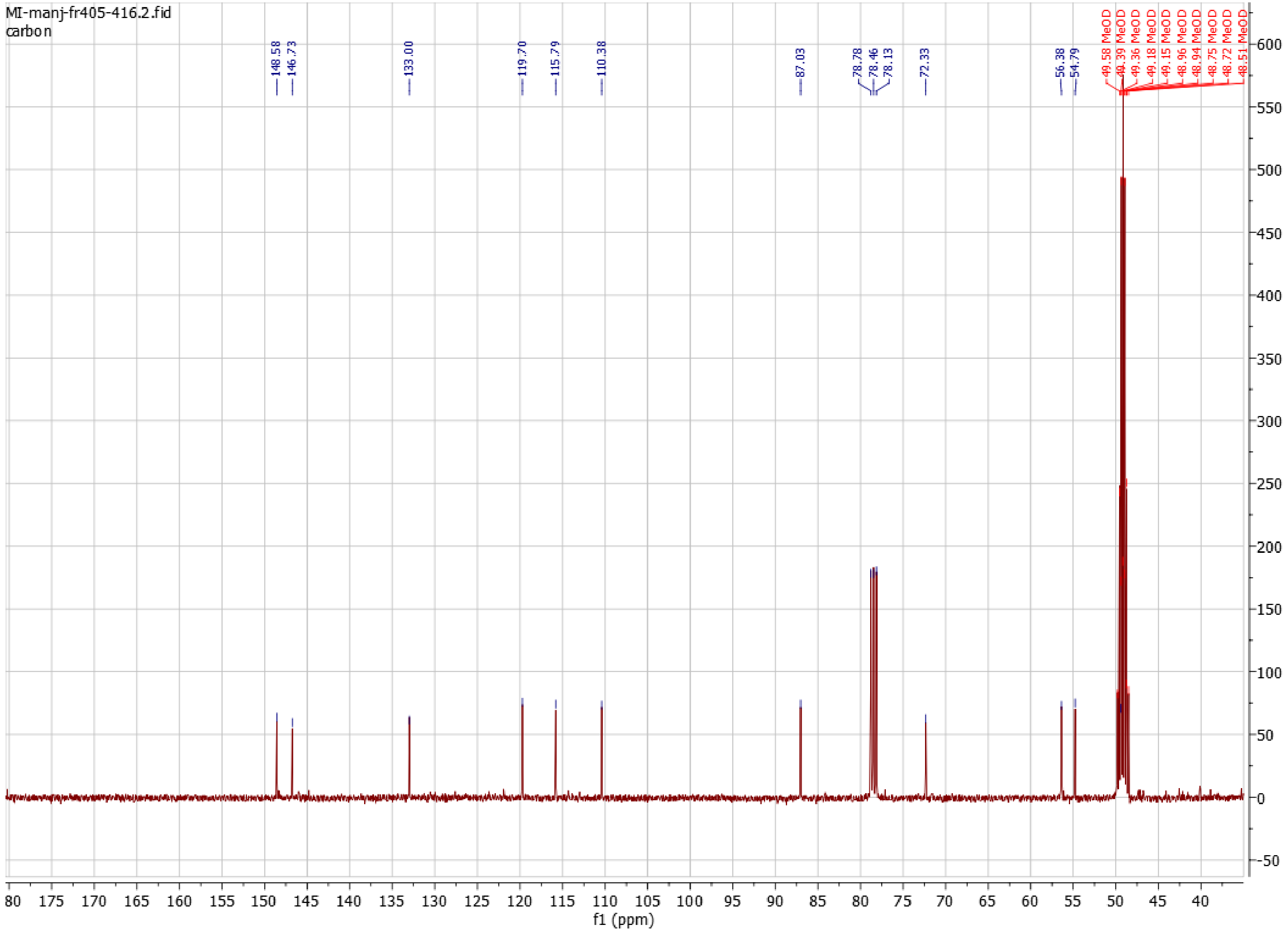

Figure S8: ^{13}C NMR spectrum of Eudesmin (4) [400 MHz, MeOH-*d4*]

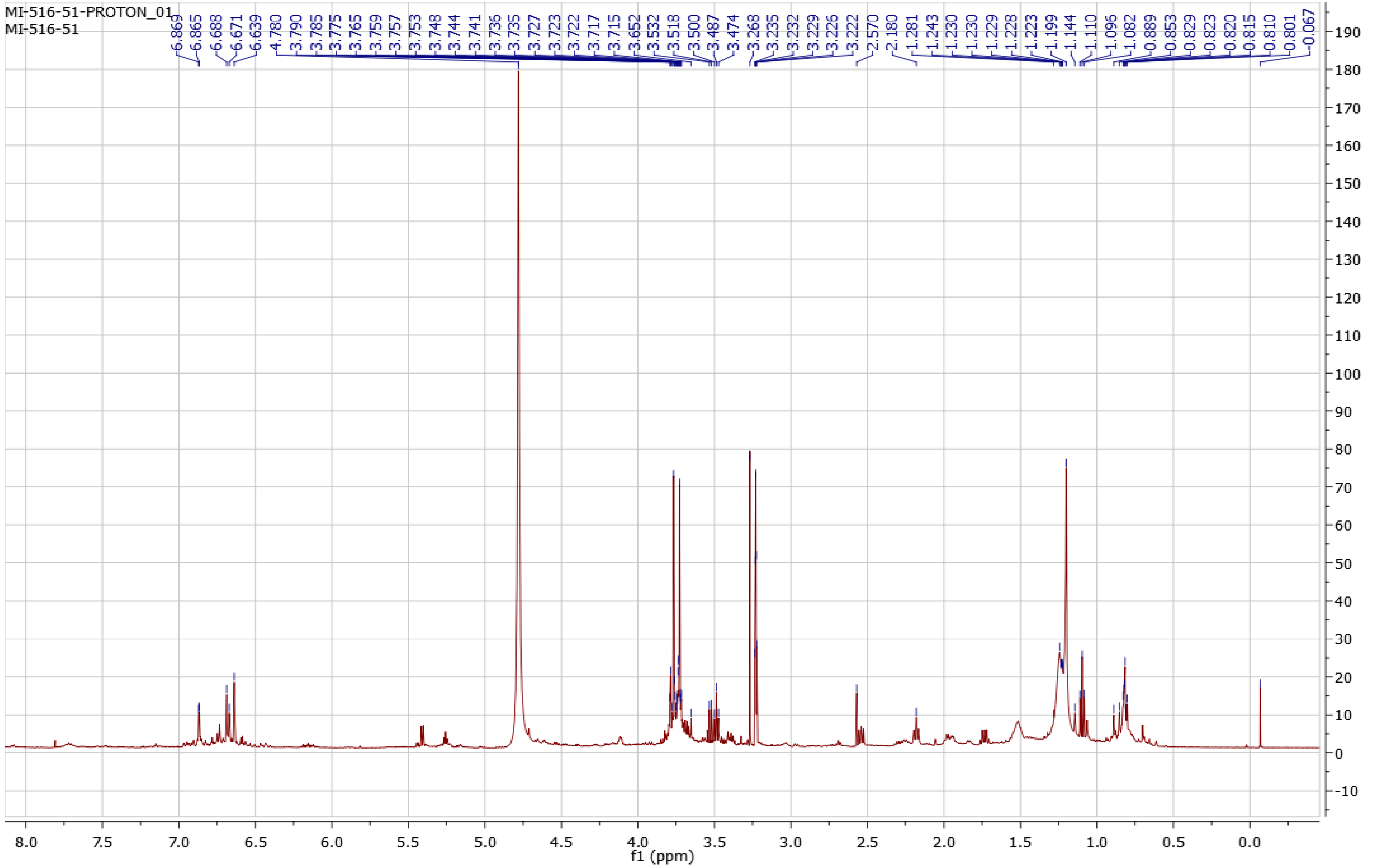

Figure S9: ^{1}H NMR spectrum of Neolignan (5) [400 MHz, MeOH-*d4*]

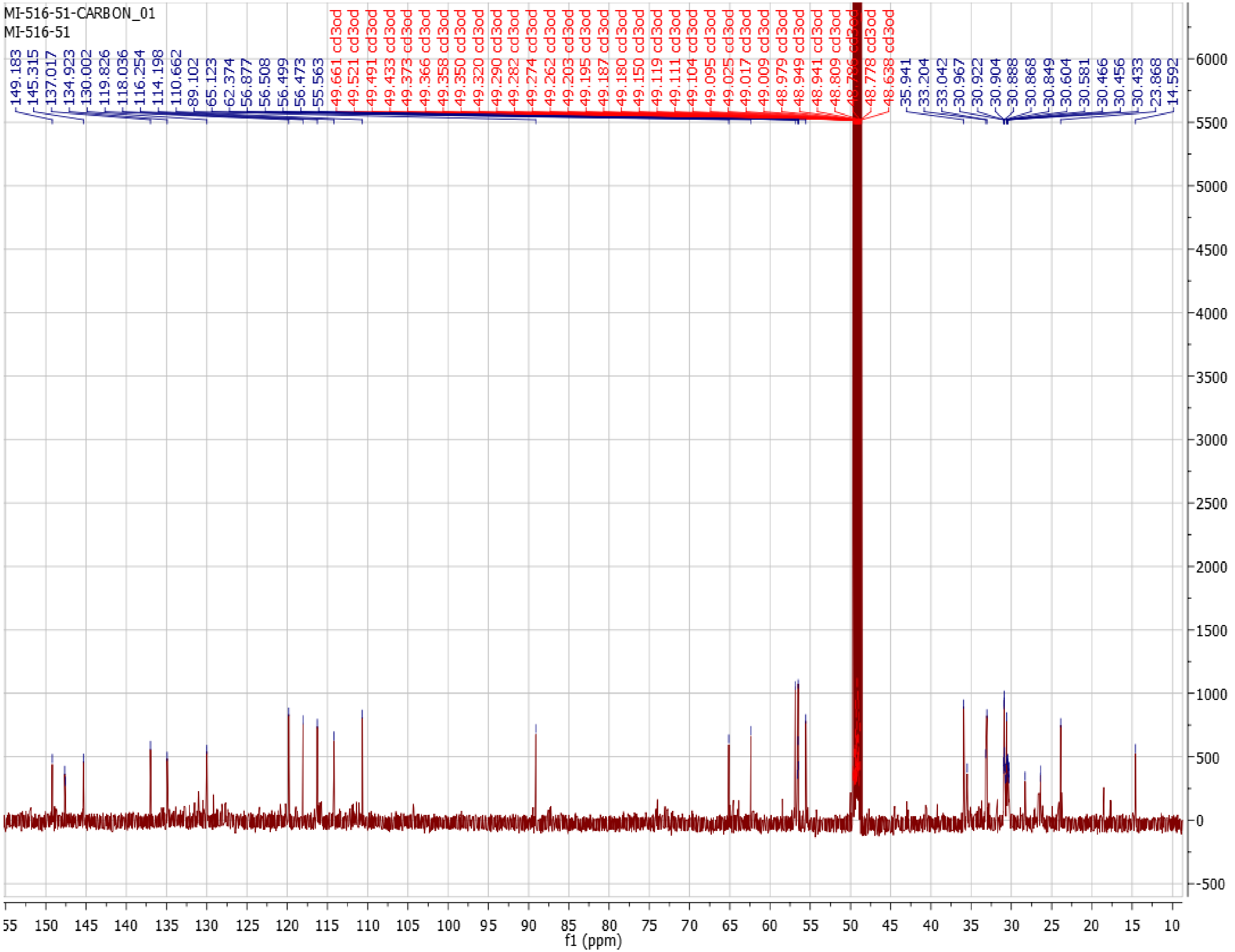

Figure S10: ^{13}C NMR spectrum of Neolignan (**5**) [400 MHz, MeOH-*d4*]

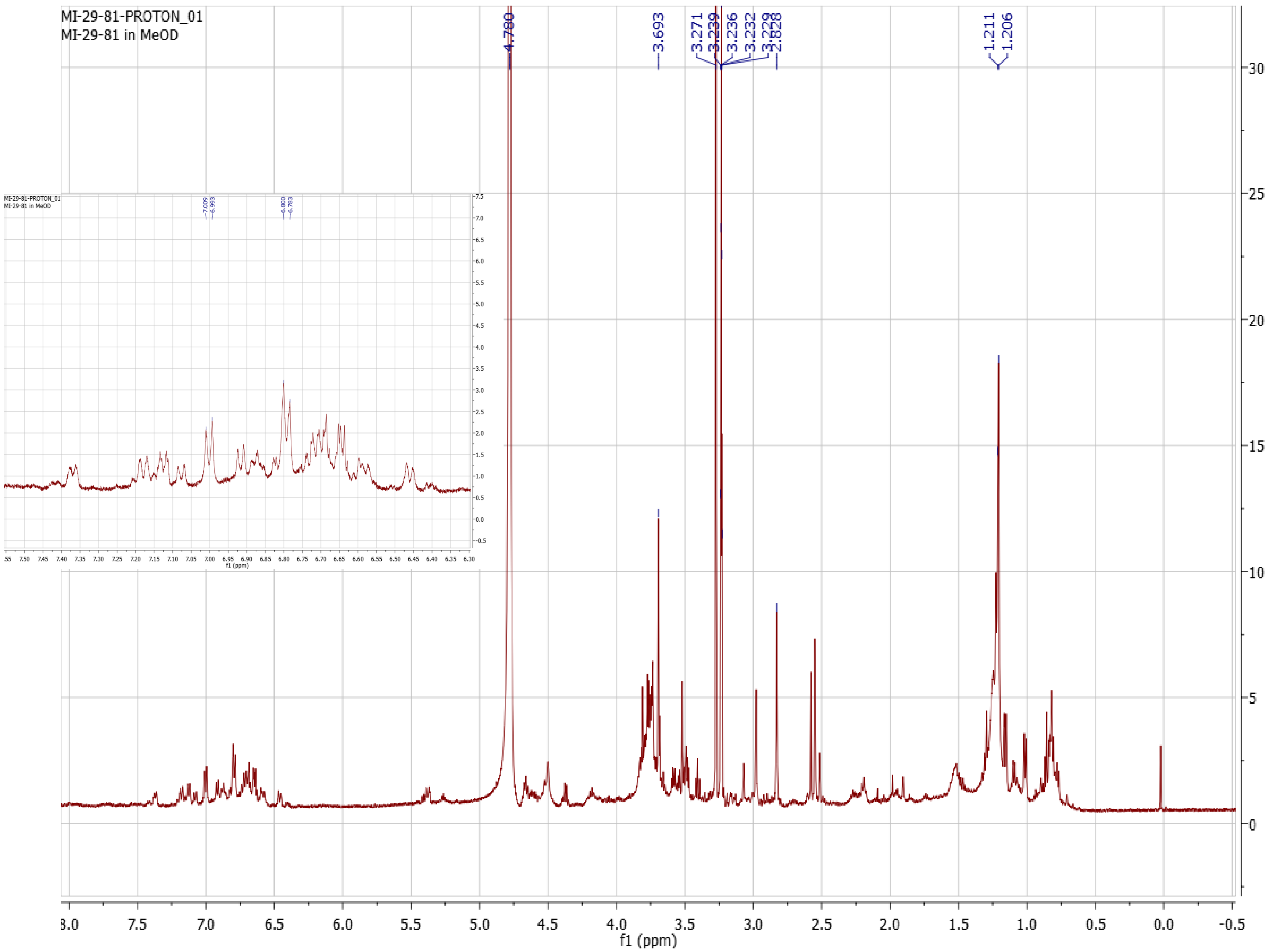

Figure S11: 1H NMR of cyclic hexapeptide mixrture; RA-V (6) and RA-XXI (9) [400 MHz, MeOH-*d4*]

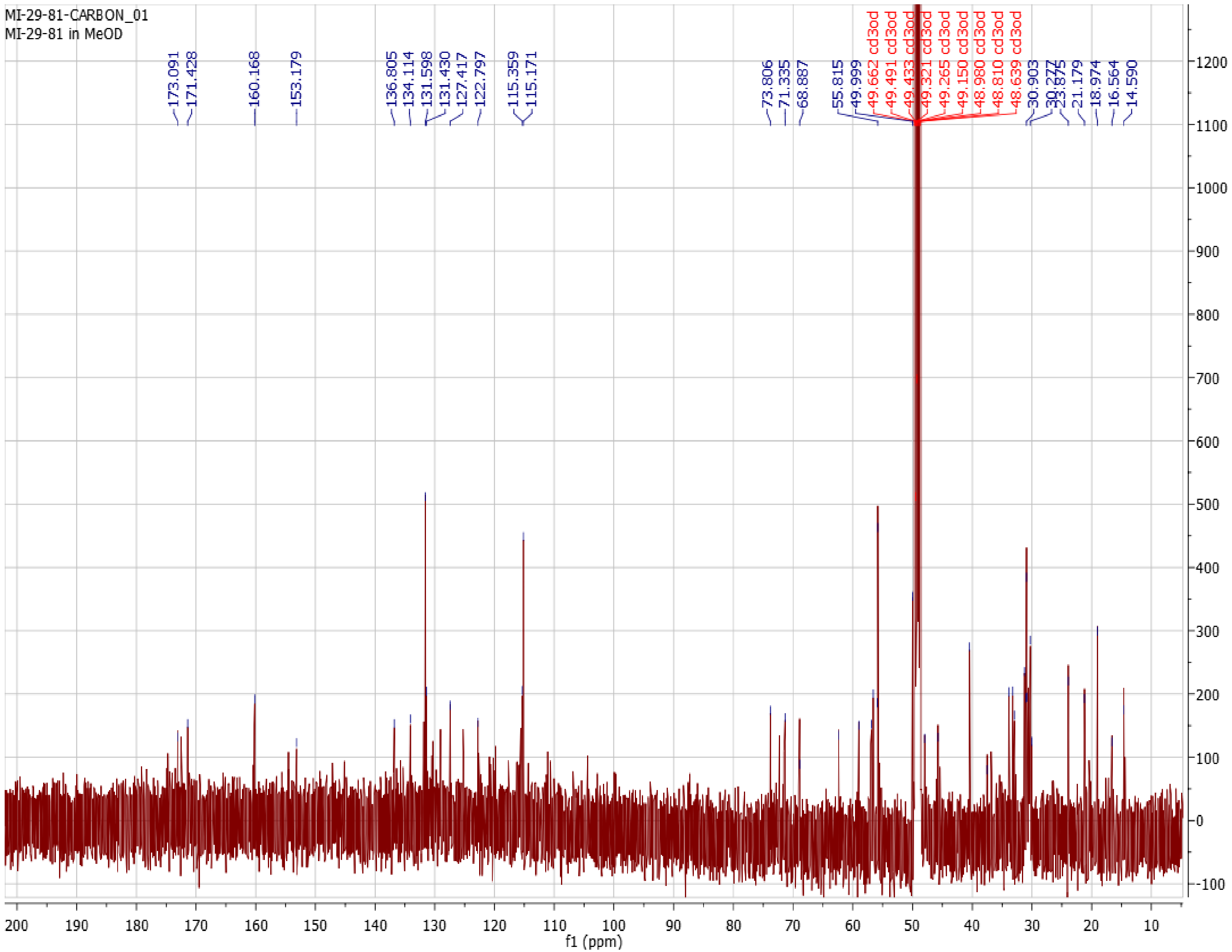

Figure S12: ^{13}C NMR of cyclic hexapeptide mixrture; RA-V (6) and RA-XXI (9) [400 MHz, MeOH-*d4*]

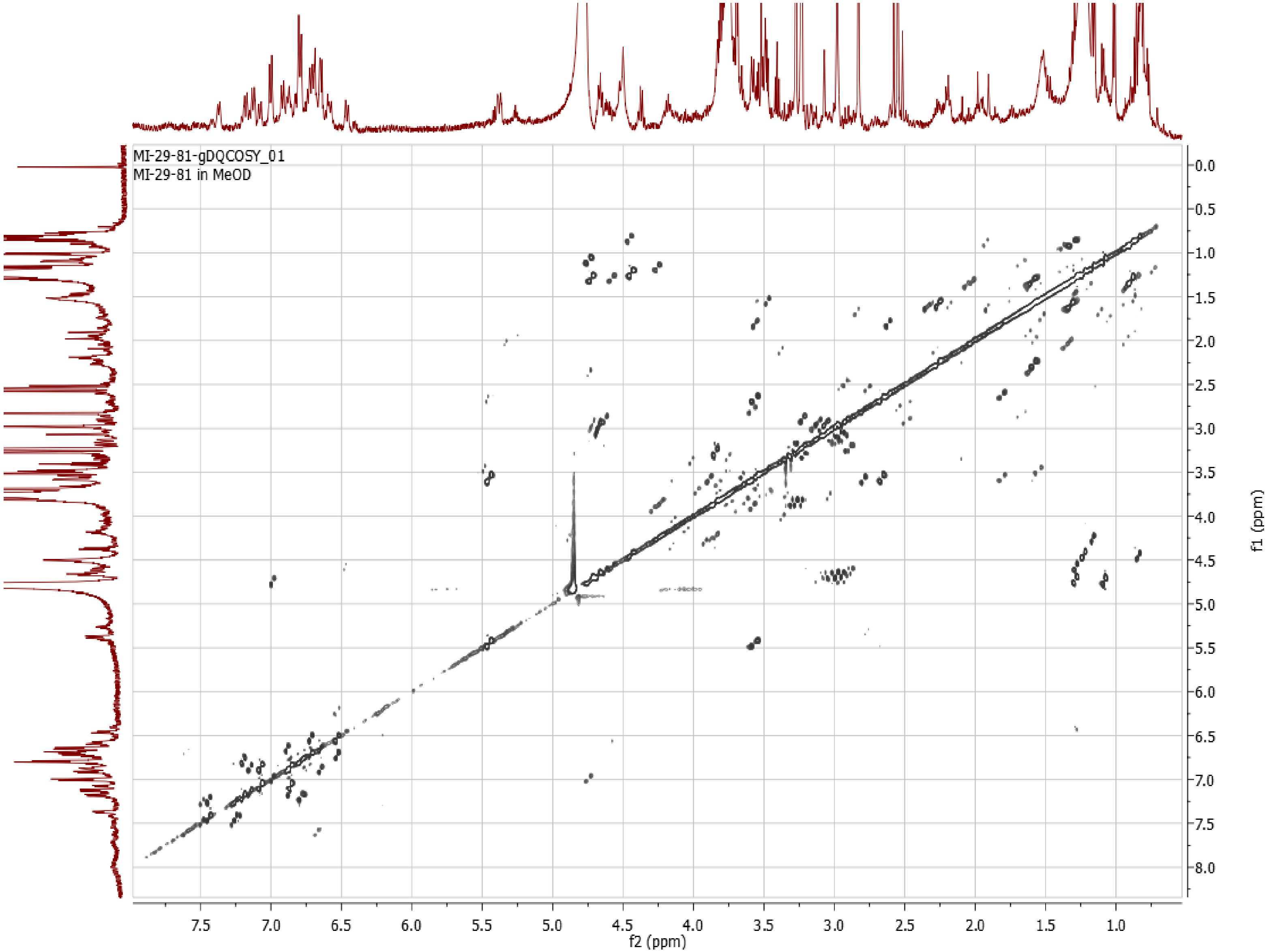

Figure S13: COSY of cyclic hexapeptide mixrture; RA-V (**6**) and RA-XXI (**9**) [400 MHz, MeOH-*d4*]

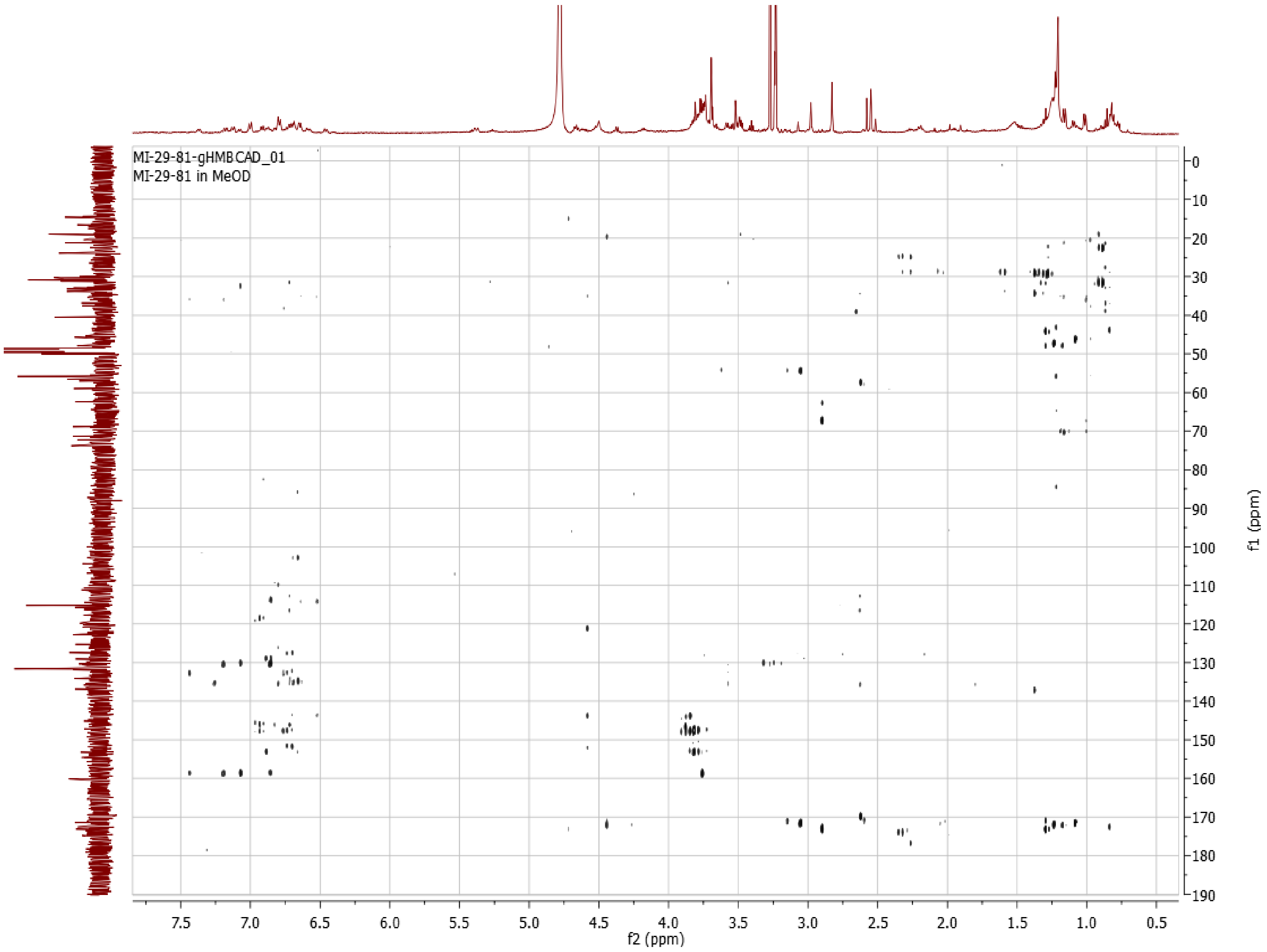

Figure S14: HMBC of cyclic hexapeptide mixrture; RA-V (6) and RA-XXI (9) [400 MHz, MeOH-*d4*]

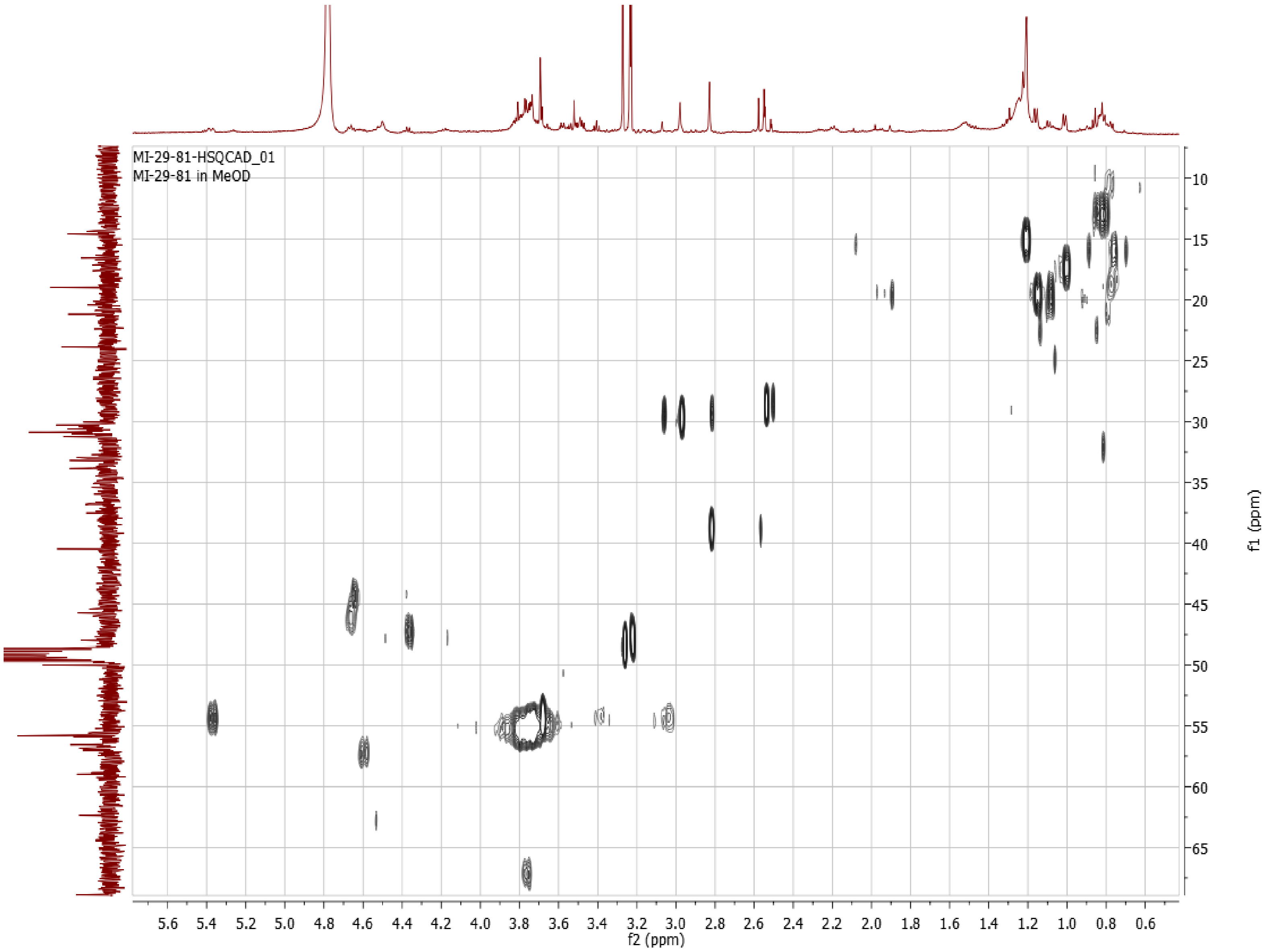

Figure S15: HSQC expansion of cyclic hexapeptide mixrture; RA-V (6) and RA-XXI (9) [400 MHz, MeOH-*d4*]

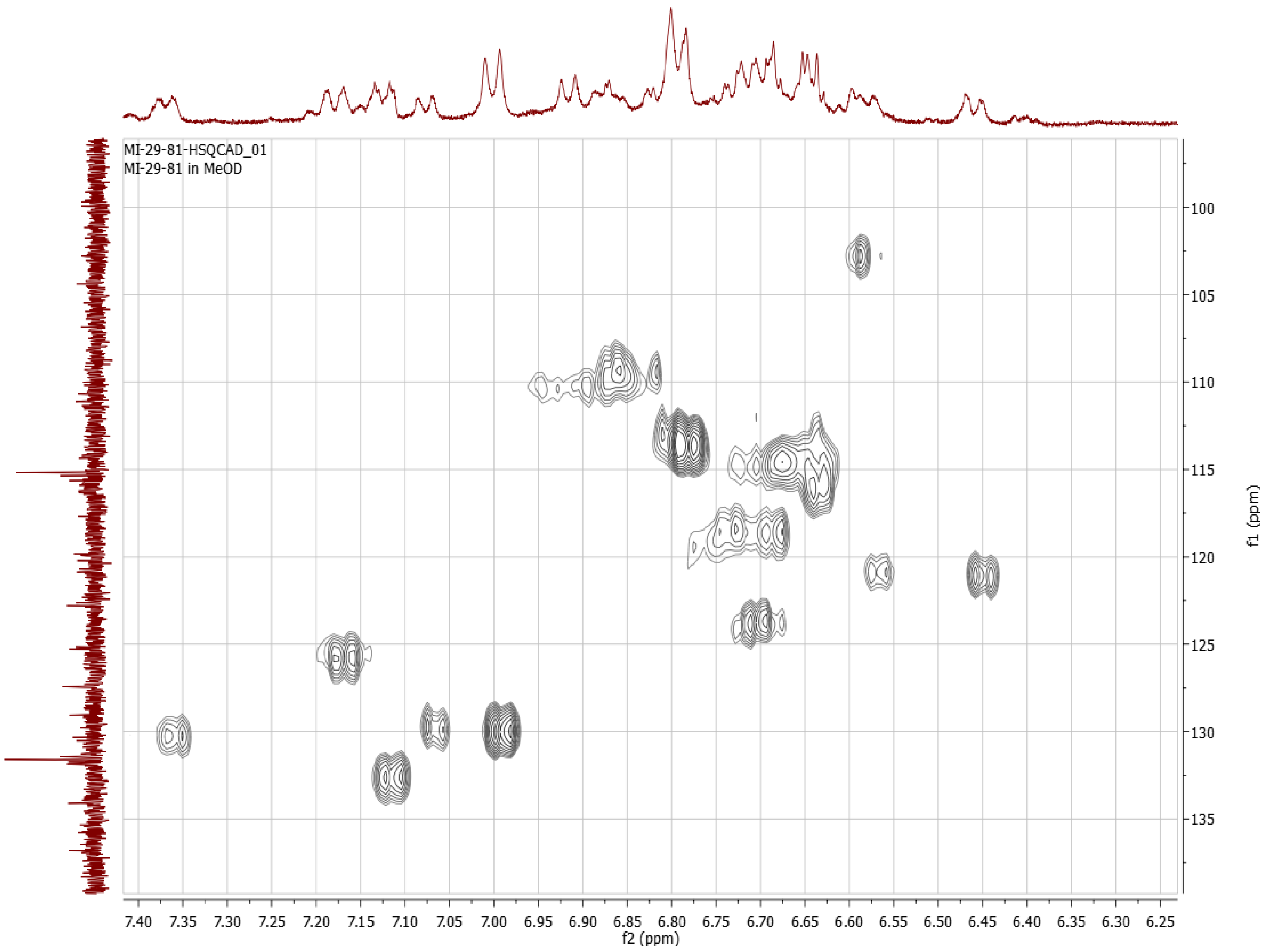

Figure S16: HSQC expansion of cyclic hexapeptide mixrture; RA-V (6) and RA-XXI (9) [400 MHz, MeOH-*d4*]

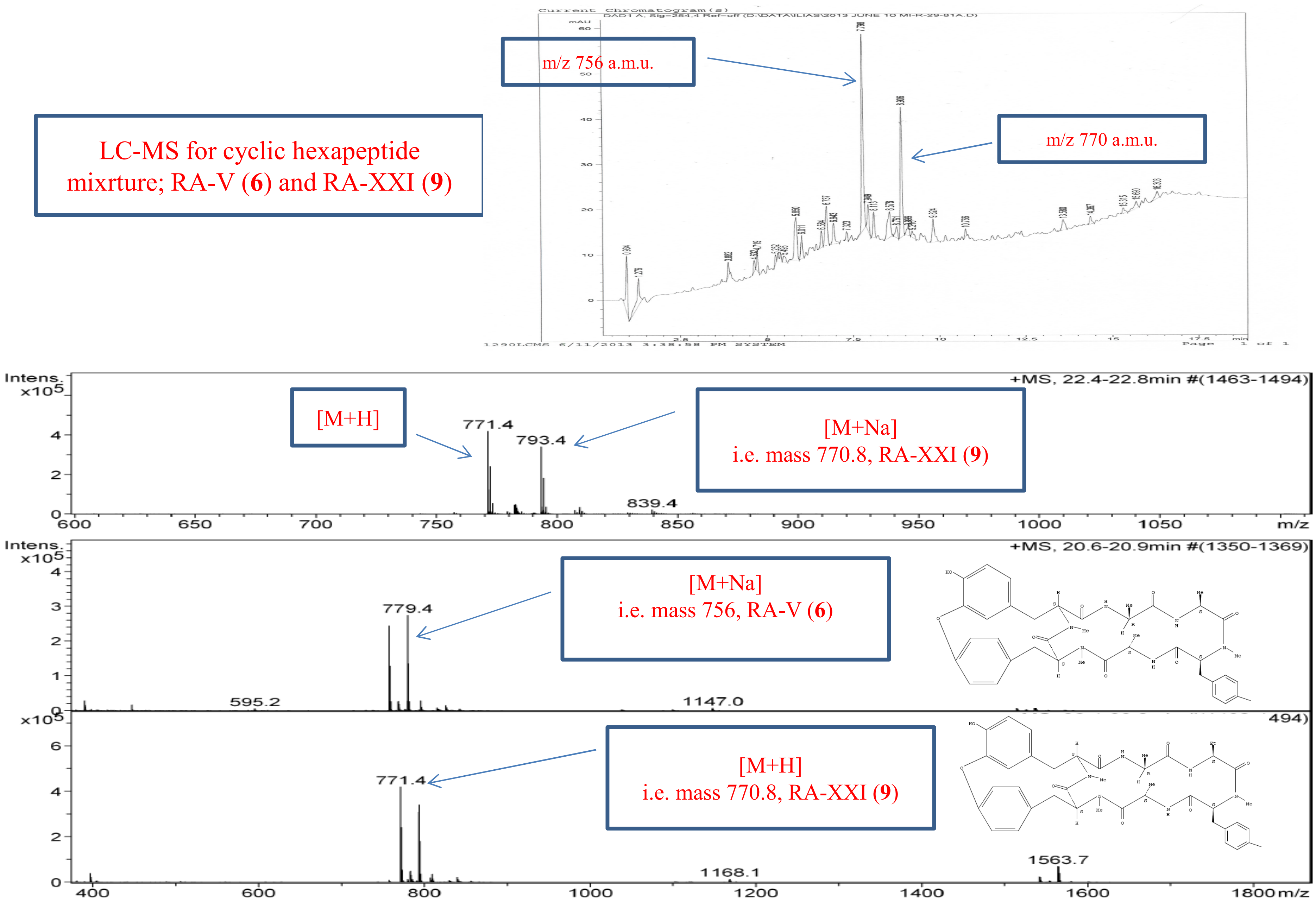

Figure S17: LC/MS spectrum of RA-V (**6**) and RA-XXI (**9**)

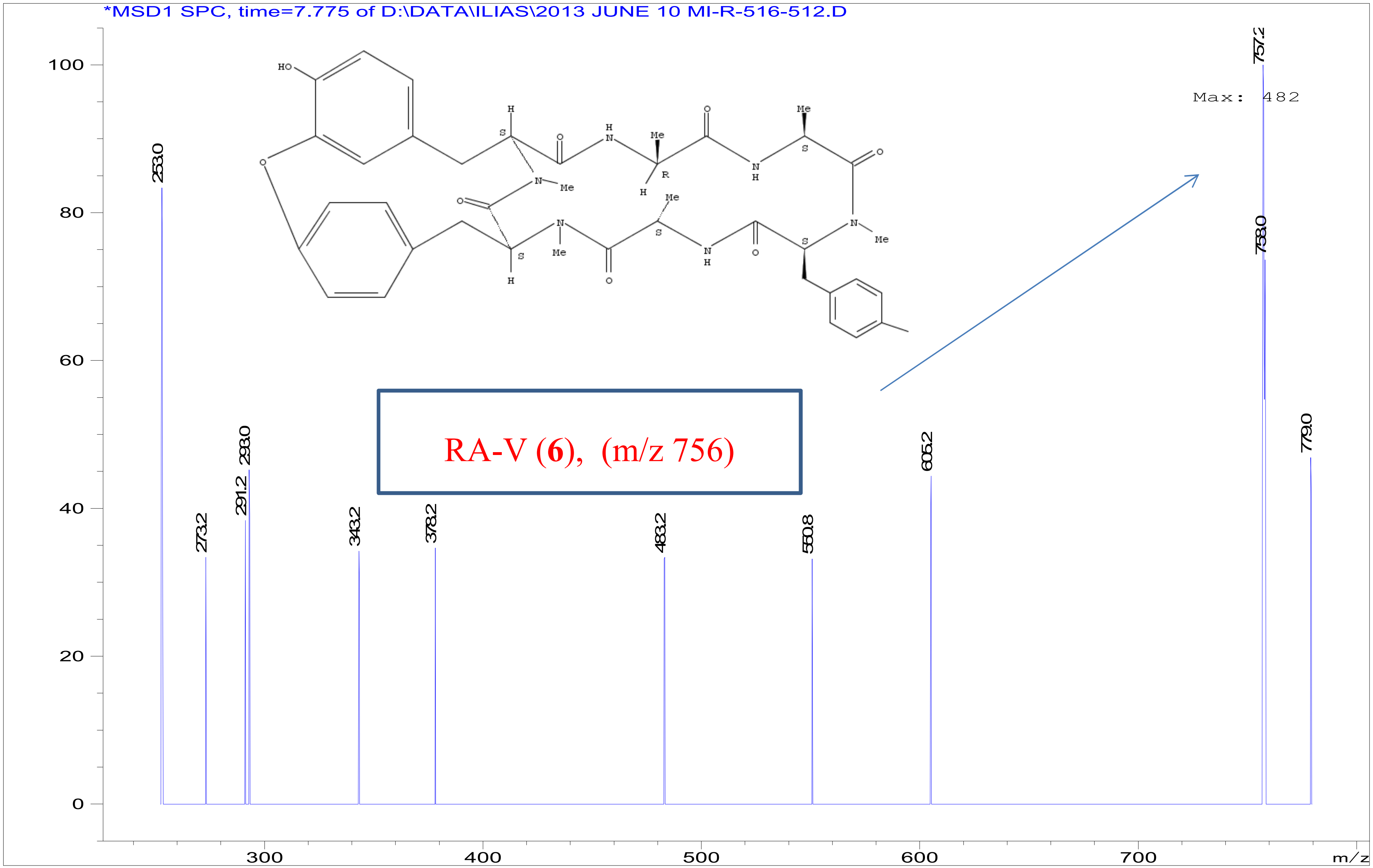

Figure S18: Mass (+) spectrum of RA-V (**6**)

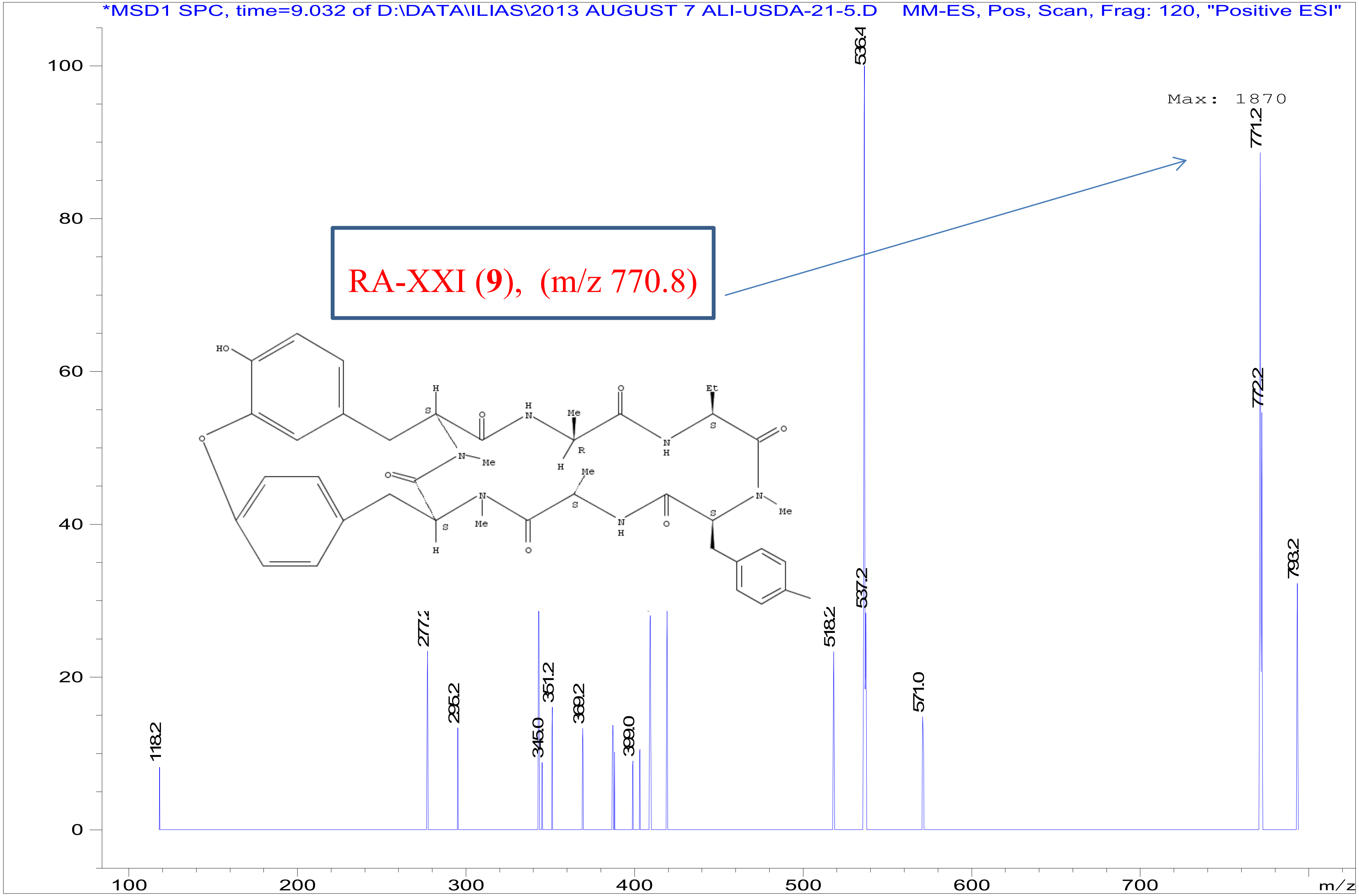

Figure S19: Mass (+) spectrum of RA-XXI (**9**)

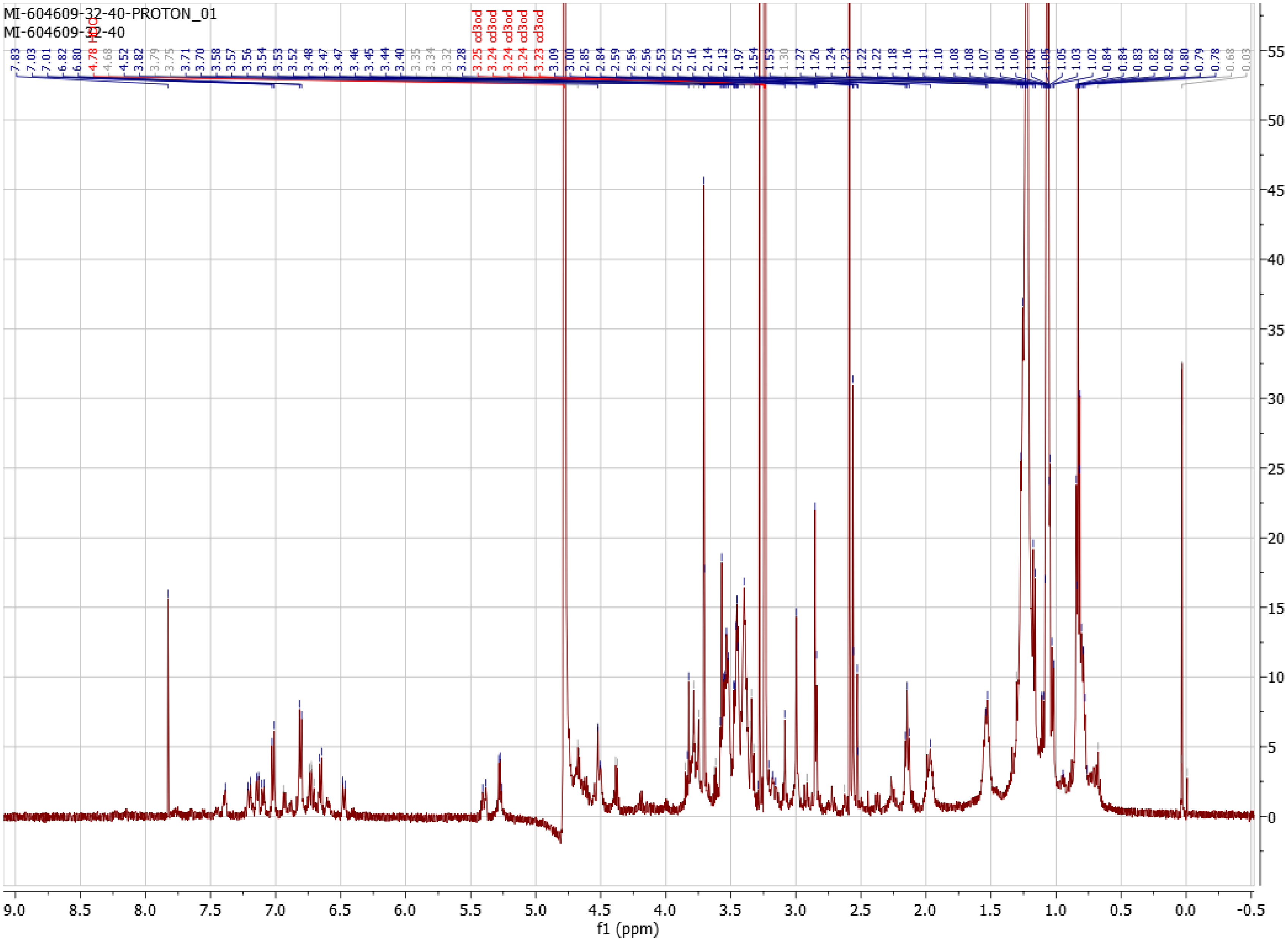

Figure S20: ^{1}H NMR of cyclic hexapeptide RA-V (6) [400 MHz, MeOH-*d4*]

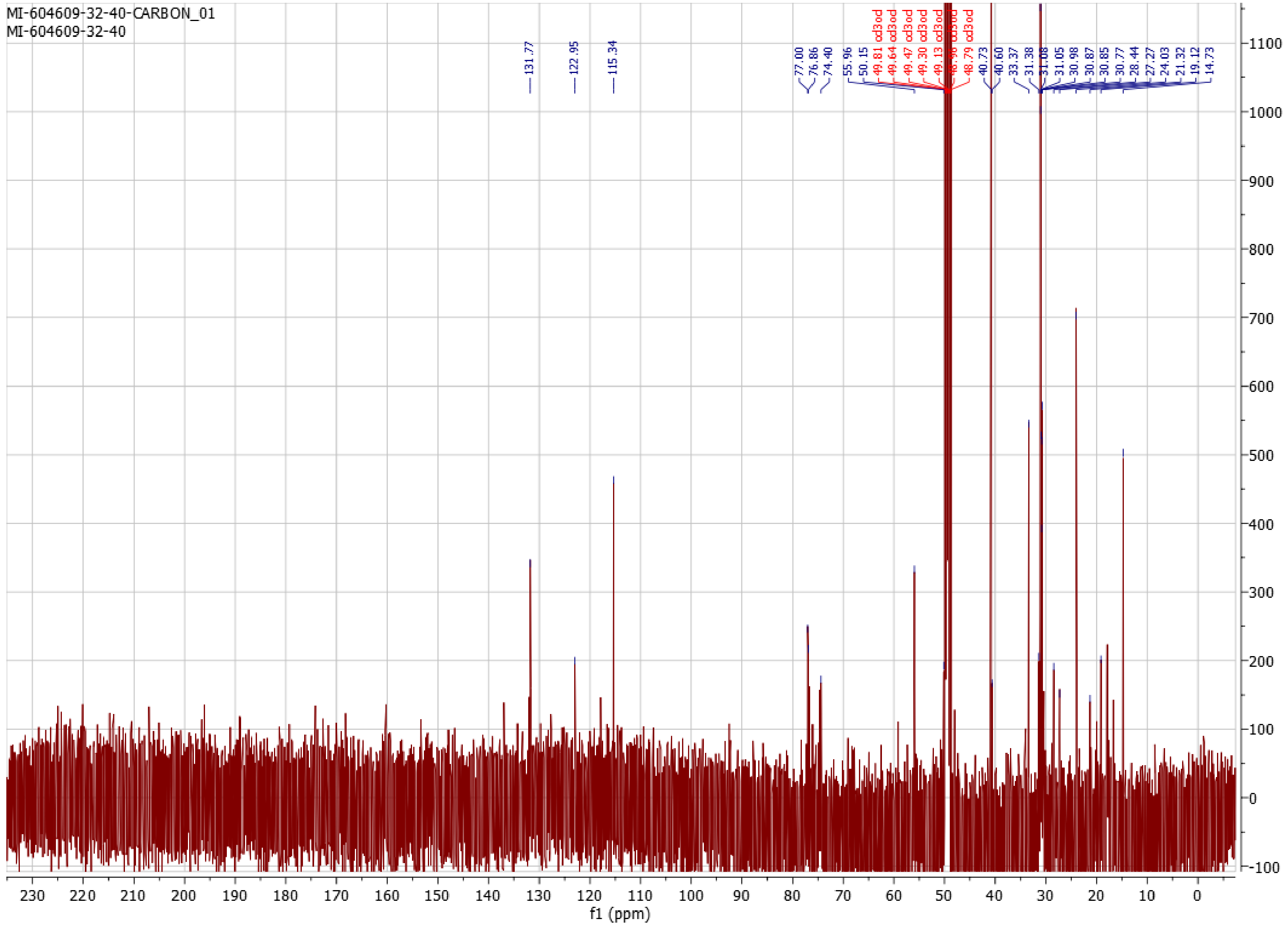

Figure S 21: ^{13}C NMR of cyclic hexapeptide RA-V (**6**) [400 MHz, MeOH-*d4*]

Table S1: Activities of Simulated Gastric Fluid (SGF) treated and untreated compound 6 against cancer related signaling pathways in Hela cells.

	Stat3/ IL-6	Smad / TGF-b	Ap-1/ PMA	NF-kB/ PMA	E2F/ PMA	Myc/ PMA	Ets/ PMA	Notch/ PMA	FoxO	Wnt/ m-wnt 3a	Hdghog/ PMA	miR21	pTK
Untreated Compound 6 (1µg/ml)	-4	33	48	33	82	-8	60	65	51	6	1	183	50
SGF treated compound 6 (1µg/ml)	-4	41	43	34	8	20	58	56	68	12	5	150	48

Values are percentage of luciferase induction at 1µg/ml concentration by the indicated inducers when compared to pTK as control. Test agents were added to the cells 30mins before the indicated inducer and were harvested for the luciferase assay four or six (Notch, FoxO, Wnt, Hedgehog and miR21) hours later. No inducer was added to the cells transfected with control vector (pTK), FoxO and miR-21.

Table S2: Details of plasmids used in transfection assays.

Pathway	Vector Name	Description	Enhancer Sequence	Source	Reference
pTK Control	TA-luc	Empty Control Reporter Vector		Affymetrix/LR0000	Zaki et al., 2013
STAT3	pSTAT3-luc	Stat3(1) Luciferase Reporter Vector	TGCTTCCCGAATTCCCGAATTCCCGAATTCCCGAATTCCCGAATTCCCGAACGT	Affymetrix/LR0077	Zaki et al., 2013
SMAD3/4	pSMAD-luc	Smad Luciferase Reporter Vector	AGTATGTCTAGACTGAAGTATGTCTAGACTGAAGTATGTCTAGACTGA	Affymetrix/LR0116	Zaki et al., 2013
AP-1	P1A1-AP1-Luc	4 copies of synthetic AP-1 binding site	TGAGTCATGAGTCATGAGTCATGAGTCA	PMID: 11689104	Zaki et al., 2013; Subbramiah et al., 2001
NF-kB	pBIIX-Luc	2 copies of of binding site from immunoglobuin K promoter	GCTACAAGGGACTTTCCGCTGGGGACTTTCCAGG	PMID: 8108136	Zaki et al., 2013; Chang et al., 1994
E2F	p1A1-E2F-Luc	2 copies of E2F Binding Site	TTTCGCGCTTTCGCGC	PMID: 11689104	Zaki et al., 2013; Subbramiah et al., 2001
Myc	pGL2M4-Luc	Minimal SV40 promoter-4 copies of Myc-Max binding site	CACGTGCACGTGCACGTGCACGTG	PMID: 9150134	Zaki et al., 2013; Laherty et al., 1997
Ets	p1A1-ETS-Luc	2 copies of inverted Ets2 binding sites	GACCGGAAGTAGTTCCGGTCGACCGGAAGTAGTTCCGGTC	PMID: 11689104	Zaki et al., 2013; Subbramiah et al., 2001
Notch	TA-CSLPromoter-Luc	2 CSL head to head binding sites upstreatm of Hes1	CAAACAAAAAATTCTTTTTCGTGAAGAACTCCAAAAAT	Signosis/ LR-2200	Zaki et al., 2013
FOXO	pFKHR-Luc	Forkhead box 01- Forkhead Cis Element	CAAAACAACAAAACAACAAAACAACAAAACAA	Signosis/LR-2037	Zaki et al., 2013
Wnt P	TOPFlash TCF Reporter Plasmid	Thymidine Kinase Minimal Promoter-6 Copies of HNF1 homeobox A-Luciferase	AGATCAAAGGGGGTAAGAKCAAAGGGGGTAAAATCAAAGGGGGCCCCCTTTGATCTTACCCCCTTTGATCTTACCCCCTTTGATCT	Millipore Sigma/21-17[illegible]	Zaki et al., 2013
Hedgehog	pGli1-Luc	GLI zinc finger transcription factor- 5 repeats of Gli1 binding site	GAAGACCACCCACAATGAAGACCACCCACAATGAAGACCACCCACAATGAAGACCACCCACAATGAAGACCACCCACAATGAAGAC CACCCACAAT	Signosis/LR-2095	Zaki et al., 2013
miR-21	pSicoR-mCherry-miR-21	Two Complimentary miR21 binding sites	GTACATCAACATCAGTCTGATAAGCTACCCGGGTCAACATCAGTCTGATAAGCTAG	PMID: 19419954	Huang et al., 2009

References:

14. Zaki MA, Balachandran P, Khan S, Wang M, Mohammed R, Hetta MH, et al. Cytotoxicity and modulation of cancer-related signaling by (Z)- and (E)-3,4,3',5'-tetramethoxystilbene isolated from Eugenia rigida. Journal of natural products. 2013;76:679-84.

28. Hamilton, K.L., Sheehan, S.A., Retzbach, E.P., Timmerman, C.A., Gianneschi, G.B., Tempera, P.J., Balachandran, P. and Goldberg, G.S., *Effects of Maackia amurensis seed lectin (MASL) on oral squamous cell carcinoma (OSCC) gene expression and transcriptional signaling pathways.* Journal of Cancer Research and Clinical Oncology, 2020(Nov): p. 1-13.
